# Rational engineering of ratiometric calcium sensors with bright green and red fluorescent proteins

**DOI:** 10.1038/s42003-021-02452-z

**Published:** 2021-07-29

**Authors:** Diming Zhang, Emily Redington, Yiyang Gong

**Affiliations:** Department of Biomedical Engineering, Duke University, Durham, NC USA

**Keywords:** Fluorescence imaging, Fluorescent proteins

## Abstract

Ratiometric genetically encoded calcium indicators (GECIs) record neural activity with high brightness while mitigating motion-induced artifacts. Recently developed ratiometric GECIs primarily employ cyan and yellow-fluorescent fluorescence resonance energy transfer pairs, and thus fall short in some applications that require deep tissue penetration and resistance to photobleaching. We engineered a set of green-red ratiometric calcium sensors that fused two fluorescent proteins and calcium sensing domain within an alternate configuration. The best performing elements of this palette of sensors, Twitch-GR and Twitch-NR, inherited the superior photophysical properties of their constituent fluorescent proteins. These properties enabled our sensors to outperform existing ratiometric calcium sensors in brightness and photobleaching metrics. In turn, the shot-noise limited signal fidelity of our sensors when reporting action potentials in cultured neurons and in the awake behaving mice was higher than the fidelity of existing sensors. Our sensor enabled a regime of imaging that simultaneously captured neural structure and function down to the deep layers of the mouse cortex.

## Abstract (web summary)

Zhang, Redington & Gong develop FRET-based ratiometric calcium sensors with pairs of green and red fluorescent proteins. Their best performers, Twitch-GR and Twitch-NR inherit the superior photophysical properties of their constituent fluorescent proteins, enabling them to outperform existing ratiometric calcium sensors in brightness, photobleaching, and signal fidelity metrics.

## Introduction

Genetically encoded calcium indicators (GECIs) are powerful tools for reporting neural activity and elucidating neural function^1–3^. Neural activity such as action potentials modulate calcium fluxes across the neuron membrane through voltage-gated calcium channels, and thus drive intracellular calcium concentration changes. Genetically encoded fluorescent sensors of calcium can effectively report such proxy calcium activity: GECIs can record neurons with large scale over thousands of neurons simultaneously^4^, with genetic targeting that isolates the dynamics of specific neuron types, and chronically over long durations. Concomitant imaging and behavioral manipulations in model organisms can develop the relationship between neural activity and neural function.

GECIs largely fall into two categories: the single fluorescence channel category of GECIs includes families of sensors such as the GCaMPs^5–7^ and RCaMPs^8–10^; the two fluorescence channel category of GECIs includes families of sensors such as the yellow cameleons (YCs)^11–14^ and Twitches^15–17^. The single-channel GECIs typically combine a calcium sensing domain with a circularly permuted fluorescent protein (cpFP); the allosteric interaction between the calcium sensing domain’s conformation and the fluorescent protein’s chromophore reports changes in the calcium concentration with changes in fluorescence intensity^6,7^. GECIs using allostery have fast kinetics, large intensity changes, and potential for multiplexing with other sensors in separate fluorescence channels; these factors have pushed single-channel GECIs to the forefront of biological investigations. While single-channel GECIs support high signal-to-noise ratio (SNR) in many imaging experiments, they could face challenges in experiments with a limited photon budget. Imaging deep into scattering tissue or imaging small sub-cellular structures could challenge these sensors, because these sensors support such large dynamic range by biasing the baseline fluorescence intensity of the sensor to a dim state^7,18^.

Two-channel ratiometric sensors can potentially overcome issues of brightness by combining a calcium sensing domain with two fluorescent proteins to report calcium activity. The sensing domain responds to changes in calcium concentration by modulating the relative positioning and fluorescence resonance energy transfer (FRET) efficiency between the two fluorescent proteins, and consequently modulating the fluorescence intensities of the two fluorescent proteins^13,17^. Because two-channel GECIs use native forms of fluorescent proteins, the sensors have similar brightness as the fluorescent proteins, and can offer three practical benefits. First, the sensors’ high brightness provides high shot-noise limited signal fidelity during dynamic imaging experiments. Second, the bright ratiometric sensors provide structural information, such as sub-cellular axonal or dendritic compartments, in conjunction with dynamic information during experiments. This ability would allow experimentalists to chronically and repeatably locate target structures for imaging. Third, because these sensors offer two bright, simultaneous measurements, the ratio between the two channels could mitigate intensity noise induced by out-of-plane motion commonplace during in vivo imaging experiments. While intensity-based sensors could also support the latter two experiments when multiplexing a second, bright fluorescent emitter in a spectrally separated channel, ratiometric sensors support such experiments using one excitation source.

The development of ratiometric GECIs lags behind the development of single-channel GECIs^19–21^. When sensing calcium after action potentials in photon-rich experimental preparations such as cell culture or shallow regions of mammalian tissue, the SNRs of the ratiometric sensors are lower than the SNRs of the single-wavelength sensors; the calcium affinity and dynamic range of ratiometric sensors are also lower than the respective metrics of single-channel sensors^6,7,17^. Further improvements of these ratiometric GECI metrics could derive from improvements in the GECI emitters. One potential area for improvement is the limited set of fluorescent proteins used within ratiometric GECIs. FRET-based calcium sensors have traditionally paired one cyan fluorescent protein with one yellow-fluorescent protein^11–17^. These pairs have high FRET efficiencies and large ratiometric responses to changes in calcium concentration, but their photophysical properties are not as strong as those of peer fluorescent protein pairs. Some weaknesses of cyan-yellow FRET pairs are the following: the cyan fluorescent protein donors are not bright compared to peer green and yellow-fluorescent protein donors^22–24^, the yellow-fluorescent protein acceptors suffer from fast photobleaching^24–26^, and both cyan and yellow-fluorescent proteins suffer from reversible photobleaching^27–31^. Cyan-fluorescent proteins are also prone to scattering because tissue scattering coefficients increase with higher photon energies; the scattering of excitation light limits the imaging depth, while the scattering of emission light reduces the photon collection efficiency and SNR^32^. To overcome these photophysical deficiencies, several FRET-based sensors have employed green and red fluorescent proteins to serve as the donor and acceptor, respectively^33–36^. While green-red FRET pairs are photostable and have potentially large theoretical Förster radii^33^, creating a high fidelity FRET-based calcium sensor using green-red pairs still requires optimizing the practical FRET efficiency of the sensor and maximizing the brightness of the sensors. To date, there does not exist a green-red FRET calcium sensor that effectively reports neuronal calcium response both in vitro and in vivo.

In this work, we engineered a set of FRET-based ratiometric calcium sensors that overcame the existing challenges listed above. Our sensors used green-fluorescent proteins as the donor and the brightest red fluorescent protein, mScarlet^37^, as the acceptor. These sensors combined the fluorescent proteins and a well-optimized calcium sensing domain from troponin C (TnC)^17^ in an alternate configuration. An iterative screening process optimized the linkers between the three sensor components. Two sensors among the resulting optimized candidates, Twitch-GR and Twitch-NR, had large fluorescence responses to calcium concentration changes and had calcium binding affinities near the neuronal calcium concentration. These sensors co-opted the high brightness and photostability of their constituent fluorescent proteins. Due to the superior brightness of green and red fluorescent proteins compared to cyan and yellow-fluorescent proteins, respectively, our sensors outperformed existing ratiometric sensors in brightness and shot-noise limited SNR. When expressed in cultured HEK cells and mammalian neurons, our sensors demonstrated high SNR when responding to changes in intracellular calcium concentration. When expressed in live mice, our sensors reported orientation-selective response from the superficial layers of the visual cortex. The high brightness and sensitivity of our sensor-enabled imaging deep into the mouse cortex, reporting function and sub-cellular structure as deep as layer 5.

## Results

### Systematic screening of a green-red ratiometric Ca^2+^ sensor optimized the FRET efficiency and folding of our sensors

Our ratiometric GECI linked a red fluorescent protein FRET acceptor, the Ca^2+^ sensing domain from TnC^17^, and a green-fluorescent protein FRET donor in order. We selected mScarlet as the acceptor because it is the brightest red fluorescent protein. We initially selected mNeonGreen (mNeon) and mCitrine as the donors because mNeon is the brightest green-fluorescent protein^24^, while mCitrine has known stable circular permutations^17^. We selected the troponin sensing domain because it undergoes a large conformational change with increased Ca^2+^ concentration; this change brings the pair of fluorescent proteins closer together and increases the FRET interaction between the pair. Compared to most peer FRET calcium sensors using the Camodulin-M13 sensing domain, FRET calcium sensors using TnC had higher affinity for Ca^2+^. Although the affinity of Twitch sensors was slightly lower than the affinity of YC-Nano sensors^13^, Twitch had a more linear response. We performed systematic screening by looping over three design steps: we expressed candidate sensors in cultured HEK293 cells, then imaged the spectra of individual cells expressing these candidates, and finally quantified the FRET efficiency of the candidate sensors and the quantum yield of the sensors’ component fluorescent proteins (Supplementary Fig. 1; “Methods”). These two metrics respectively served as proxies for the sensors’ sensitivity to Ca^2+^ and maturation.

Iterative looping over these three steps improved the calcium sensitivity and folding efficiency of four sensor configurations: mNeon-TnC-mScarlet, mScarlet-TnC-mNeon, mNeon-TnC-cpScarlet, and mScarlet-TnC-cpCitrine (Supplementary Fig. 2a). These configurations tested whether the circular permutations or the relative placements of the donor and acceptor impacted the FRET efficiency of the sensor. We noticed that the calcium sensitivity of configurations employing one circularly permuted fluorescent protein (mNeon-TnC-cpScarlet and mScarlet-TnC-cpCitrine) was higher than the calcium sensitivity of configurations employing only non-circularly permuted fluorescent proteins (mNeon-TnC-mScarlet and mScarlet-TnC-mNeon) (Supplementary Fig. 2b). However, sensors using the circular permutation of mScarlet had low acceptor quantum yield (Supplementary Fig. 2c, d). Sensors using the circular permutation of mCitrine had high donor and acceptor quantum yield, and simultaneously produced a large calcium response (Supplementary Fig. 2d–f).

**Fig. 1:**
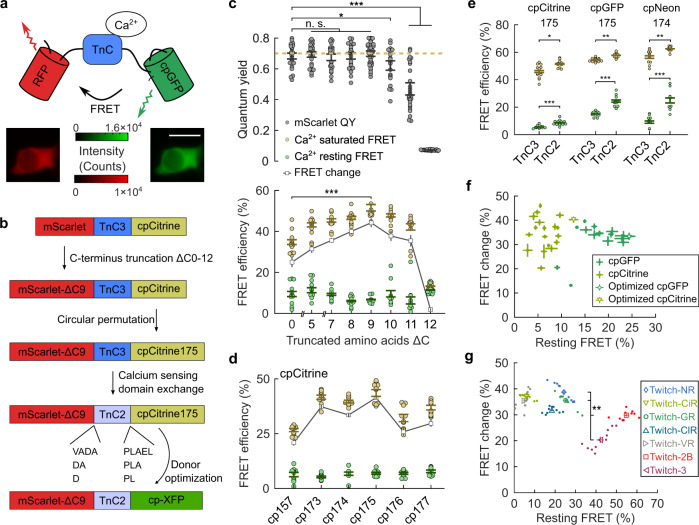
Rational design on the fluorescent proteins and linkers created sensors with high Ca^2+^ sensitivity. **a** Schematic representation of the sensor construct linking the RFP (mScarlet), calcium sensing domain (TnC), and circularly permuted GFP-analog in series. The two inset images represent the fluorescence intensity in the two imaged channels from one example cell. The illumination intensity was 5 mW/mm^2^. The scale bar is 20 µm. **b** A schematic illustration shows various sensor designs that maximized on the Ca^2+^ sensitivity after improvements through amino acid truncation, circularly permutation, sensing domain optimization, linker optimization, and donor optimization. The XFP referred to a set of fluorescent proteins including mNeonGreen, mCitrine, EGFP, mClover3, or mVenus. **c** The FRET efficiency of the sensor and the quantum yield of the acceptor remained high for truncations of mScarlet shorter than 9 amino acids. Larger truncations not only extinguished the red fluorescence, but also significantly reduced the FRET efficiency between the green and red fluorescent proteins. The horizontal dash represents the reported quantum yield of native mScarlet. (**d**) Circular permutation of the mCitrine donor at specific locations optimized the FRET efficiency change between the sensors’ Ca^2+^-resting state and Ca^2+^-saturated state. **e** Sensors using the TnC2 calcium sensing domain had significantly higher Ca^2+^-saturated FRET efficiency (*yellow*) and the Ca^2+^-resting state FRET efficiency (*green*) than those of sensors using the TnC3 calcium sensing domain. **f** The relationship between the resting-Ca^2+^ FRET efficiency of sensors and the change in FRET efficiency for various linkers between sensing domains and fluorescent proteins. The optimal linker for each donor (*open triangular and circular symbols*) had both high resting FRET efficiency and high FRET efficiency change. **g** The relationship between the various FP pairs’ resting FRET efficiency and change in FRET efficiency between Ca^2+^-resting and Ca^2+^-saturated states. Dots represent FRET metrics measured from individual cells, while the symbols represent the mean FRET metrics of each variant. All error bars are s.e.m., *n* ≥ 10 cells for each sensor. All statistical tests were the two-sided Wilcoxon rank-sum test, where * signifies *p* < 0.1, ** signifies *p* < 0.01, *** signifies *p* < 0.001, n.s. signifies not significant.

These preliminary results pushed us to focus on the sensor configuration that employed mScarlet as the acceptor and a circularly permuted green-fluorescent protein-analog as the donor (Fig. 1a). We expressed sensor candidates with this configuration in cultured HEK cells while engineering four sensor features: the truncation of the mScarlet C-terminus, the circular permutation of the donor, the TnC calcium sensitivity, and the linkers between the TnC and fluorescent proteins (Fig. 1b). First, we truncated the mScarlet C-terminus to increase the FRET efficiency between the sensor’s two fluorescent proteins. We found that sensors with truncations up to 9 amino acids retained red fluorescence when expressed in HEK cells. The acceptor quantum yields of these sensors were not statistically different from the quantum yield of native mScarlet, near 0.7 (*p* > 0.44 for cuts of 5–9 amino acids, two-sided Wilcoxon rank-sum test, *n* ≥ 10 cells for each sensor). Increasing the number of truncated amino acids from 0 to 9 significantly increased the FRET efficiency change between the Ca^2+^-resting and Ca^2+^-saturated states (*p* = 2.5 × 10^−4^, two-sided Wilcoxon rank-sum test, *n* ≥ 10 cells for each sensor) (Fig. 1c). Truncations larger than 9 amino acids significantly diminished the red fluorescence and the mScarlet quantum yield (*p* = 0.06, 1.1 × 10^−6^, and 1.4 × 10^−9^ for cuts of 10, 11, and 12 amino acids, respectively, two-sided Wilcoxon rank-sum test, *n* ≥ 10 cells for each sensor) (Fig. 1c, Supplementary Fig. 3), likely because the proximity of mScarlet to other sensor components prevented proper folding.

Second, we optimized the FRET efficiency of the sensor in the Ca^2+^-saturated state by aligning the dipole orientation between the fluorescent proteins. We attempted multiple orientations between the proteins by circularly permuting the mCitrine donor at multiple locations in the loops outside of the main fluorescent protein barrel. We quantified the brightness of these cpCitrine variants when integrated into the sensors and found that these proteins had similar brightness as circularly permuted mCitrine reported in the Twitch-3 sensor^17^ (Supplementary Fig. 4a). The cpCitrine175 variant in our sensor simultaneously maximized the FRET efficiency in the Ca^2+^-saturated state and the FRET efficiency change between the Ca^2+^-resting and Ca^2+^-saturated states (Fig. 1d).

Third, we maximized the calcium sensing domain’s sensitivity. We employed the two previously reported variants of the calcium sensing domain in TnC, TnC3, and TnC2, derived, respectively, from the Twitch-3 and Twitch-2B sensors^17^, as the sensing domains of our sensors candidates. Both the FRET efficiency in the Ca^2+^-saturated state and the FRET efficiency change between the Ca^2+^-resting and Ca^2+^-saturated states of the sensors using the TnC2 sensing domain were significantly higher than the corresponding metrics of the sensors using the TnC3 sensing domain (*p* < 7.7 × 10^−4^ and *p* < 0.017 for the Ca^2+^-resting and Ca^2+^-saturated state comparison, two-sided Wilcoxon rank-sum test, *n* ≥ 10 cells for each sensor) (Fig. 1e), matching the previous reports^17^. We thus focused on using the TnC2 sensing domain within our optimized sensor.

Fourth, we optimized the linkers between the fluorescent proteins and the TnC sensing domain. We quantified the baseline FRET and FRET efficiency change between the Ca^2+^-resting and Ca^2+^-saturated states for 19 linker variants within the mScarlet-TnC2-cpCitrine configurations (Fig. 1f; Supplementary Table 1). The variant that maximized the baseline FRET was mScarlet-ΔC9-DA-TnC2-PLA-cpCitrine175, hereafter called Twitch-CiR.

Using Twitch-CiR as a foundation, we further integrated other stable circularly permuted green-fluorescent proteins, such as cpGFP^38^, cpVenus^12^, cpClover^33^, and cpNeon^39^, into our sensor configuration as the donor. We optimized these sensors by engineering the same four regions of the sensors (Fig. 1e–g, Supplementary Figs. 4–6). We named the resulting optimized sensors as Twitch-GR (mScarlet-ΔC9-DA-TnC2-PLA-cpGFP175), Twitch-VR (mScarlet-ΔC9-DA-TnC2-PLA-cpVenus175), Twitch-ClR (mScarlet-ΔC9-DA-TnC2-PLA-cpClover175), and Twitch-NR (mScarlet-ΔC9-DA-TnC2-PLA-cpNeon174) (Supplementary Table 2). Among all optimized variants, Twitch-GR and Twitch-NR had the largest FRET efficiencies in the Ca^2+^-resting state and large changes in FRET efficiency between the Ca^2+^-resting and Ca^2+^-saturated states (Fig. 1g). While the FRET efficiencies in the Ca^2+^-resting state of Twitch-GR and Twitch-NR were smaller than that of Twitch-2B and Twitch-3, the changes in the FRET efficiency of Twitch-GR and Twitch-NR were significantly larger than that of Twitch-2B and Twitch-3 (*p* < 1.3 × 10^−3^, two-sided Wilcoxon rank-sum test, *n* ≥ 10 cells for each sensor).

### Green-red fluorescent Twitch sensors detected changes in calcium concentration with high fidelity in cultured mammalian cells

**Fig. 2:**
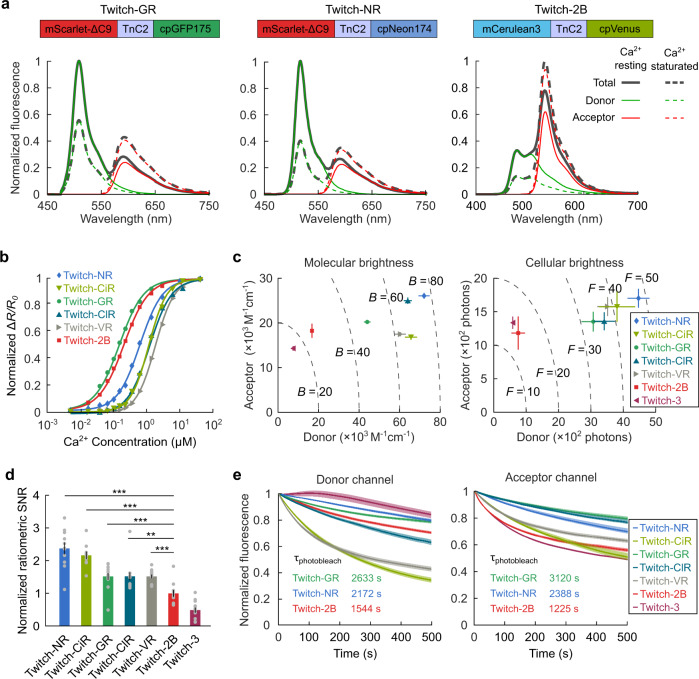
A palette of optimized green-red fluorescent GECIs displayed high Ca^2+^ sensitivity, brightness, and photostability. **a** Average spectra of Twitch-GR, Twitch-NR, and Twitch-2B in Ca^2+^-resting and Ca^2+^-saturated states (*n* ≥ 10 cells). **b** Ca^2+^ titration curves for Twitch-GR, Twitch-NR, Twitch-CiR, Twitch-ClR, Twitch-VR, and Twitch-2B. Solid lines are linear regressions of the data to a first-order Hill model. **c** The molecular (*left*) and cellular (*right*) brightness of various sensor candidates calculated from spectral measurements and dual-view images, respectively (error bars are s.e.m., *n* ≥ 10 cells for each sensor in the molecular brightness test and *n* ≥ 100 cells for each sensor in the cellular brightness test). Dashed lines denote isocontours of equal total molecular brightness or total cellular brightness. **d** The ratiometric SNR of various green-red sensor candidates when changing from the Ca^2+^-resting state to the Ca^2+^-saturated states were significantly higher than the ratiometric SNR of Twitch-2B (error bars are s.e.m.; ** signifies *p* < 0.01, *** signifies *p* < 0.001, two-sided Wilcoxon rank-sum test; *n* ≥ 10 cells for each sensor). The SNRs were normalized to the average SNR of Twitch-2B. Gray dots indicate individual data points. **e** Average photobleaching curves of various sensor candidates expressed in HEK293 cells in the resting state, separated into donor (*left*) and acceptor (*right*) fluorescence channels (shaded regions are s.e.m, *n* ≥ 30 cells for each sensor). The illumination intensity was 5 mW/mm^2^.

We refined the analysis of our five green-red FRET sensors’ calcium response when expressed in mammalian cells and when expressed as purified protein. We expressed the sensors in HEK293 cells and measured their spectra in the Ca^2+^-resting state and Ca^2+^-saturated state. The spectra of all five sensors showed decreases in the donor fluorescence and increases in the acceptor fluorescence with increasing calcium concentration (Fig. 2a; Supplementary Fig. 5). We also recorded the spectra of the sensors as purified proteins using Ca^2+^ titration (Fig. 2b; “Methods”). Fits to these titration curves found the Ca^2+^ affinity (*K*_*d*_) of Twitch-GR as 150 nM; Twitch-GR was more calcium-sensitive than Twitch-2B, which had a *K*_*d*_ of 200 nM (Supplementary Table 3). In general, sensors with the TnC2 calcium sensing domain were more sensitive than sensors with the TnC3 calcium sensing domain (Supplementary Fig. 6). Because the basal calcium concentration of neurons is ~50–100 nM^40^, we focused on Twitch-GR and Twitch-NR for further characterization and application in neuron imaging.

We next assessed the brightness of the sensors when expressed in mammalian cells and expressed as purified protein. We first integrated portions of the spectra from individual HEK cells to compute the molecular brightness of the sensors (Fig. 2c, left; “Methods”). The donor molecular brightness of our sensors were approximately 2–6 times the donor molecular brightness of the existing Twitch sensors; this ratio was commensurate with the relative brightness between the green-fluorescent donors of our sensors and the cyan-fluorescent donors of the existing Twitch sensors. The acceptor channels of our sensors were as bright as the acceptor channels of the existing Twitch sensors; this ratio was commensurate with the relative brightness between the mScarlet acceptor of our sensors and the yellow-fluorescent acceptors of the existing Twitch sensors. We also quantified the brightness of the donor and acceptor fluorescence channels when the sensors were expressed in individual HEK cells (Fig. 2c, right; “Methods”). In the HEK cell Ca^2+^-resting state, the fluorescence intensities of the donor channels of our sensors were about 3–5 times the fluorescence intensities of the donor channels of the existing Twitch sensors.

We calculated the sensors’ shot-noise limited ratiometric SNR by combining the fluorescence intensities of the red and green-fluorescent components with the measured ratiometric change between the Ca^2+^-resting and Ca^2+^-saturated states (Fig. 2d; “Methods”). Due to their superior brightnesses, all five red-green sensors outperformed Twitch-2B and Twitch-3 in the ratiometric SNR (*p* < 4.5 × 10^−3^, two-sided Wilcoxon rank-sum test, *n* > 10 cells for each sensor) (Fig. 2d). Among green-red FRET sensors, Twitch-NR and Twitch-CiR significantly outperformed the other three sensors (*p* = 3.0 × 10^−4^, 6.0 × 10^−4^, and 8.3 × 10^−4^ when comparing Twitch-NR to Twitch-GR, Twitch-ClR, and Twitch-VR, respectively; *p* = 3.7 × 10^−4^, 1.2 × 10^−3^, and 2.3 × 10^−4^ when comparing Twitch-CiR to Twitch-GR, Twitch-ClR, and Twitch-VR, respectively; two-sided Wilcoxon rank-sum test, *n* > 10 cells for each sensor).

Finally, we quantified the photostability of the various sensors when expressed in HEK cells (Fig. 2e, Supplementary Table 3). Twitch-GR and Twitch-NR were the most photostable sensors; their donor photobleaching time constants were respectively 1.7 and 1.4 times as long as the donor photobleaching time constant of Twitch-2B, while their acceptor photobleaching time constants were respectively 2.5 and 1.9 times as long as the acceptor photobleaching time constant of Twitch-2B. These large differences between the photobleaching rates of our green-red sensors and the rates of Twitch sensors were commensurate with the difference between the photobleaching rate of the component fluorescent proteins in our sensors and the component fluorescent proteins in Twitch-2B^24,41^. We also observed transient reverse photobleaching of the cyan fluorescent protein donor within Twitch-3 (Fig. 2e, Supplementary Fig. 7), a previously observed phenomenon^28–30^.

### Green-red fluorescent Twitch sensors reported action potentials from cultured neurons with high SNR

**Fig. 3:**
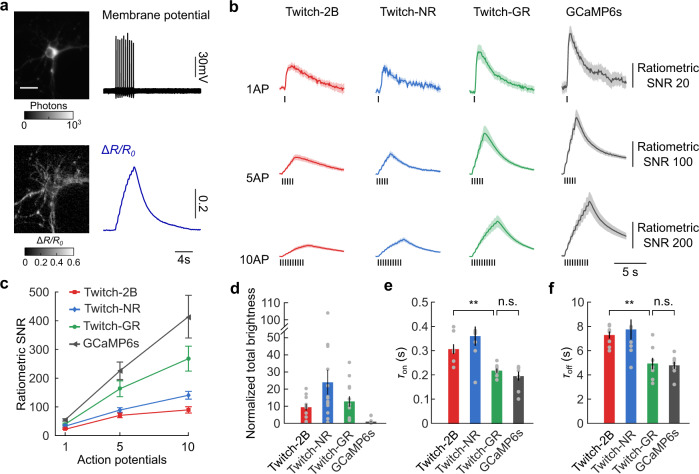
Twitch-GR and Twitch-NR sensors accurately detected action potentials in cultured hippocampal neurons. **a** The total brightness of a cultured neuron expressing Twitch-GR (*top left*) along with the spatial Δ*R/R* response (*bottom left*). The action potentials within the electrical recording of this neuron (*top right*) matched the ratiometric transients within the optical recording of this neuron (*bottom right*). The scale bar is 30 µm. **b** The average ratiometric SNR response from neurons expressing Twitch-2B, Twitch-GR, Twitch-NR, and GCaMP6s when firing one, five, and ten action potentials, respectively. Solid lines and transparent color regions indicate the mean ± s.e.m. (*n* ≥ 10 neurons for each sensor). Black dashes indicate the times of the electrical stimulation. **c** The relationship between the peak ratiometric SNR and the number of the action potentials from neurons expressing Twitch-2B, Twitch-GR, Twitch-NR, and GCaMP6s (error bars are s.e.m., *n* ≥ 10 neurons for each sensor). **d** The total brightness of neurons at rest expressing Twitch-2B, Twitch-GR, Twitch-NR, and GCaMP6s, all normalized to the mean brightness of GCaMP6s neurons (error bars are s.e.m., *n* ≥ 10 cells for each sensor). Gray dots indicate individual data points. **e** The on- and (**f**) off-time constants of Twitch-2B, Twitch-GR, Twitch-NR, and GCaMP6s (error bars are s.e.m.). The on- and off-kinetics of Twitch-GR were significantly less than those of Twitch-2B (two-sided Wilcoxon rank-sum test, *n* = 8 cells for each sensor, ** signifies *p* < 0.01, n.s. signifies not significant). Gray dots indicate individual data points.

We assessed the response of Twitch-GR and Twitch-NR to action potentials in cultured hippocampal neurons. Simultaneous electrical recordings via loose-patch and optical recordings via dual-channel imaging showed that the fluorescence from individual channels and the ratio of the channels responded to action potentials (Fig. 3a). Twitch-2B, Twitch-GR, and Twitch-NR all responded to single action potentials with fluorescence decreases in the donor channel and fluorescence increases in acceptor channel (Supplementary Fig. 8). We quantified the response of the sensors to one, five, and ten action potentials using two metrics of SNR that simultaneously capture sensors’ sensitivity and brightness within shot-noise limited measurements: the first “ratiometric SNR” metric quantified the conventional shot-noise limited response of the ratio between the two fluorescence channels, while the second “total SNR” metric quantified the aggregate SNR when summing the response from the two channels in quadrature^42^ (“Methods”). When using the ratiometric SNR measure, Twitch-GR had SNRs that were 1.8−, 2.3−, and 3.0-times the SNRs of Twitch-2B when responding to one, five, and ten action potentials, respectively; Twitch-NR had SNRs that were 1.5-, 1.3-, and 1.6-times the SNRs of Twitch-2B when responding to those same three electrical waveforms, respectively (Fig. 3b, c). When using the total SNR measure, Twitch-GR had SNRs that were 1.8-, 2.2-, and 2.8-times the SNRs of Twitch-2B, while Twitch-NR still had SNRs that were 1.4-, 1.3-, and 1.6-times the SNRs of Twitch-2B when responding to those same three electrical waveforms, respectively (Supplementary Fig. 9). The version of Twitch-GR using the TnC3 sensing domain had a low Ca^2+^ affinity; the SNR of the TnC3 version of Twitch-GR was lower than the SNR of the TnC2 version of Twitch-GR (Supplementary Fig. 10). Our Twitch sensors obtained similarly high SNR at the periphery of the neuron (Supplementary Fig. 11). Twitch-GR approached the SNR of single-channel GECIs as well, as Twitch-GR had ~0.6 of the SNR of GCaMP6s when responding to one, five, and ten action potentials (Fig. 3c). The performance of our Twitch sensors derives partly from the high brightness of the sensors. The brightness of Twitch-GR and Twitch-NR expressed in neurons was approximately 2 times that of Twitch-2B, and more than 10 times that of GCaMP6s (Fig. 3d).

We quantified the kinetics of the sensors by fitting the single action potential response of cultured neurons to first-order exponential dynamics (Supplementary Fig. 12; “Methods”). Overall, Twitch-GR had significantly faster kinetics than Twitch-2B (*p* < 1.1 × 10^−3^ when comparing on-kinetics, *p* < 1.9 × 10^−3^ when comparing off-kinetics, two-sided Wilcoxon rank-sum test, *n* = 8 cells for each sensor) (Fig. 3e, f); the rise time (*τ*_on_) of Twitch-GR was 0.71 the rise time of Twitch-2B (Fig. 3e), while the decay time (*τ*_off_) of Twitch-GR was 0.68 the decay time of Twitch-2B (Fig. 3f). The kinetics of Twitch-GR were not statistically different from the kinetics of GCaMP6s (*p* = 0.72 and 0.88 for on- and off-kinetics, respectively, two-sided Wilcoxon rank-sum test, *n* = 8 neurons for each sensor; Fig. 3e, f).

### Twitch-GR reported structure and function from shallow and deep regions of the mouse cortex during awake behavior

**Fig. 4:**
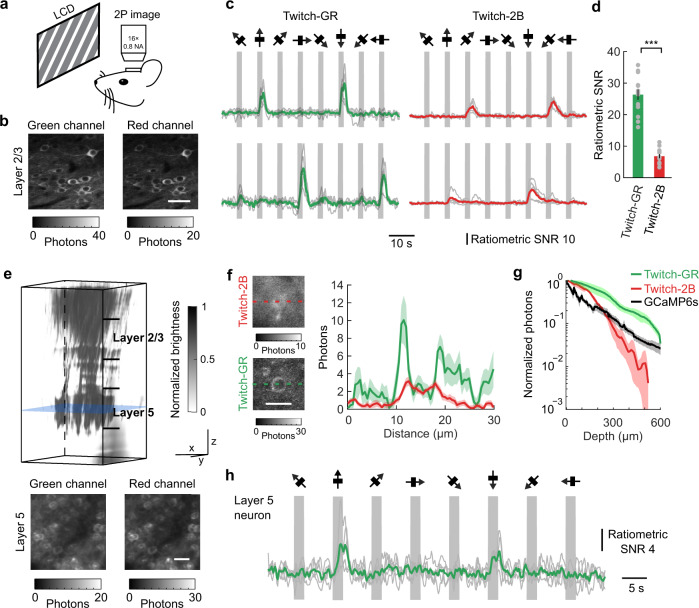
Twitch-GR reported cellular visual response profiles in mice during awake behavior. **a** The schematic shows the experimental setup for the delivery of drifting grating visual stimuli and simultaneous two-photon imaging. **b** Two-photon images from in layer 2/3 neurons expressing the Twitch-GR sensor, separated into the green (*left*) and red (*right*) fluorescence channels. The scale bar is 20 µm. **c** Ratiometric traces of representative layer 2/3 neurons expressing Twitch-GR (*left*) and Twitch-2B (*right*) displayed orientation-selective response to a set of eight different orientations of drifting grating stimuli. **d** The peak ratiometric SNR of Twitch-GR was significantly higher than the response of Twitch-2B (two-sided Wilcoxon rank-sum test, *n* = 10 neurons for each sensor, error bars are s.e.m.; *** signifies *p* < 0.001). Gray dots indicate individual data points. **e** Three-dimensional stacks imaged from the brain surface down to 600 μm depth. The *z* stack (*top*) showed the entire cortical structure, while the green and red channel images at 500 μm indicated by the blue slice of the stack (*bottom*) showed individual neurons. The three-dimensional scale bars for the top panel are 100 μm along each of the three axes, while the scale bar for the bottom panels is 30 µm. **f** *Left*: Total fluorescence images of a representative neuron expressing Twitch-2B or Twitch-GR at a depth of ~350 µm. Each image was the average of 10 imaging frames. *Right*: Profiles along the dashed lines demonstrated cytoplasmic localization for Twitch-GR, but not for Twitch-2B (shaded regions are s.e.m., *n* = 10 frames). The intensity profiles removed the background fluorescence. The scale bar is 15 µm. **g** The average number of photons normalized for excitation power obtained over the whole field-of-view as a function of the imaging depth (error bars are s.e.m., *n* = 3 stacks imaging from 2–3 mice for each sensor). (**h**) Ratiometric responses of a layer 5 neuron expressing Twitch-GR displayed orientation-selective response to eight different orientations of drifting grating stimuli.

We next assessed the performance of Twitch-GR in vivo during two-photon imaging of awake mice processing visual stimuli. We selected Twitch-GR for this imaging application because GFP has a larger two-photon response than mNeon^43^. We expressed Twitch-GR in the mouse primary visual cortex (V1) using adeno-associated viral vector mediated gene expression (“Methods”). We imaged transduced neurons using two-photon microscopy while delivering drifting grating visual stimuli of various orientations (Fig. 4a, b). In layer 2/3 of V1, Twitch-GR and Twitch-2B both reported neuronal calcium activity with representative orientation selectivity (Fig. 4c). The shot-noise limited ratiometric SNR of Twitch-GR was significantly higher than that of Twitch-2B (*p* = 1.1 × 10^−5^, two-sided Wilcoxon rank-sum test, *n* = 10 neurons for each sensor) when normalizing for excitation power (Fig. 4d). The higher SNR of Twitch-GR was partially derived from the high brightness of Twitch-GR (Supplementary Fig. 13). Similar to measurements in cultured neurons, the kinetics of Twitch-GR imaging was significantly faster than the kinetics of Twitch-2B during in vivo imaging (*p* = 1.4 × 10^−3^ and 2.0 × 10^−4^ when comparing *τ*_on_ and *τ*_off_, respectively, two-sided Wilcoxon rank-sum test, *n* ≥ 13 neurons) (Supplementary Table 4).

Twitch-GR also reported both structure and function from layer 5 neurons, deep within the V1 cortical column. The high brightness of Twitch-GR supported high shot-noise limited imaging fidelity. The long excitation and imaging wavelengths used with Twitch-GR supported deep tissue penetration. When imaging a mouse expressing Twitch-GR, an image stack from the surface of the brain to 600 μm deep into the mouse cortex showed layer 2/3 neuron somas, layer 5 neuron somas, and apical dendrites of layer 5 neurons projecting through layer 2/3 (Fig. 4e). The Twitch-2B image stack contained layer 2/3 neurons and apical dendrites of layer 5 neurons, but did not clearly show somas of neurons in layer 5 (Supplementary Fig. 13). Near the deepest regions of layer 2/3, approximately 350 μm from the surface of the brain, images of neurons expressing Twitch-GR showed that sensors were localized to the neuron cytoplasm by the fused nuclear-export tag^44^ (Fig. 4f). In contrast, images of neurons expressing Twitch-2B at the same depth showed blurred fluorescent patterns, likely because the excitation light at 860 nm used for Twitch-2B imaging experienced more scattering than the excitation at 930 nm used for Twitch-GR imaging (Fig. 4f and Supplementary Fig. 15). In addition, experiments imaging Twitch-2B through a similar volume of brain tissue demonstrated that Twitch-2B fluorescence decreased more rapidly with depth than Twitch-GR fluorescence (Fig. 4g). The superior brightness of Twitch-GR enabled the recording of orientation-selective responses to drifting grating stimuli from layer 5 neurons (Fig. 4h). Parallel experiments with GCaMP6s demonstrated similar results: GCaMP fluorescence decreased sharply with increased depth, and required higher power to reach similar SNR deep into the cortex (Supplementary Fig. 16).

## Discussion

In this work, we engineered a set of green-red ratiometric GECIs. These sensors inherited favorable photophysical properties from recently developed green and red fluorescent proteins. In turn, our sensors were brighter and more photostable than existing cyan-yellow ratiometric GECIs. The combination of bright fluorescent proteins and a recently optimized calcium sensing domain displayed high calcium affinity, comparable to the affinities of single-channel GECIs. The superior photophysical properties and calcium sensitivity translated to high SNR in vitro and in vivo neuron imaging. When imaging cultured neurons and cortical neurons in awake, behaving mice, our sensors reported calcium transients with SNR that was approximately triple the SNR of transients reported by Twitch-2B. The high brightness of our Twitch-GR enabled the imaging of orientation-selective response down to layer 5 of the visual cortex, 450–600 µm from the surface of the mouse brain. Both in vitro and in vivo, Twitch-GR was faster than existing ratiometric calcium sensors. The faster kinetics could improve the temporal resolution of the neural recordings and better associate the timing of neural activity with behavioral stimuli.

The performance of our GECIs was slightly worse than the performance of single-channel GECIs such as GCaMP6s. While our ratiometric GECIs would not be the first choice when imaging accessible regions of the brain or large structures such as neuron somas, our sensors are an attractive alternative when imaging deep structures or sub-cellular compartments. For example, we observed that our sensor provided an order of magnitude more fluorescence than Twitch-2B when imaging layer 5 of the mice cortical structure at equal excitation power. Our sensor also outperformed the GCaMP sensors at such depths at equal excitation power; such performance will likely enable imaging experiments deeper into the cortex when limited by excitation-induced tissue heating^45^. We also demonstrated that we can image the apical dendrites of the layer 5 neurons and relay activity at the periphery of cultured neurons. Our sensor thus can simultaneously provide structural information and calcium dynamics for a select class of experiments that wish to monitor both at sub-cellular compartments; one example experiment in this class is the examination of synaptic plasticity at the dendrite or dendritic spines^46,47^. Likewise, our sensors have qualitatively similar performance metrics, such as brightness, affinity, and response per action potential, as those of established ratiometric synthetic dyes, such as fura-2^48,49^. However, genetically encoded tools are likely to find applications both in vitro and in vivo due to the existence of vectors that deliver proteins to specific neural populations in live animal preparations.

Further optimization of our green-red Twitch sensors would likely improve the sensitivity and FRET efficiency of our variants during in vitro and in vivo imaging experiments^42^. The FRET efficiency will improve from the engineering of two sensor features: directly improving the component fluorescent proteins, or improving the linkers between the sensing domain and fluorescent proteins. First, improvements in the green-red FRET efficiency will arise from improvements to the GFP-analog donor quantum yield and RFP-analog extinction coefficient. Improvements in such metrics will likely arise from the continuum of improvements to fluorescent proteins over time^33,50,51^. The recent development of red fluorescent proteins targeted for FRET applications^52^ will also further improve the performance of our green-red GECIs. Second, improvements to the sensitivity of FRET sensors will arise from further improving the relative orientation of the FRET pair. For example, recent structure analysis suggested that modifications to a fluorescent protein’s β-strands near the sensing domain increased the FRET efficiency of the sensor^53^. Such mutations at the analogous positions within our green-fluorescent donors could further improve the sensor response.

Beyond calcium sensors, our work can also influence the development of other FRET-based ratiometric sensors. Our sensor employed an alternate configuration with a circularly permuted donor fluorescent protein at the C-terminus of the sensor; this strategy improved the sensor response compared to existing classes of sensors using only native green and red fluorescent proteins and existing classes of sensors using a circularly permuted acceptor. Our engineering strategies that optimized the relative placement and the relative orientation of the fluorescent protein pair on the sensor can expand the search space for any FRET-based sensor using green and red fluorescent proteins. These approaches could improve the dynamic range of FRET-based sensors of potassium ions and protein kinase activity that also employ green-red fluorescent protein pairs^33,35,52,54,55^. New sensors that employ our fluorescent protein configuration will take advantage of the green-red fluorescent FRET pairs’ inherent advantages compared to cyan-yellow-fluorescent FRET pairs; these sensors would thus enable imaging experiments with low phototoxicity, high FRET efficiency response, and deep tissue penetration in live animal preparations^50^.

## Methods

### Plasmid construction

We constructed all sensors by polymerase chain reaction (PCR) cloning. The template for the mScarlet moiety of the protein fusions originated from pmScarlet_C1 (#85042, Addgene). The template for the Troponin C (TnC) moieties originated from Twitch-2B (#100040, Addgene) and Twitch-3 (#49532, Addgene). The sfGFP, mNeon, mCitrine, mVenus, and mClover moieties of the fused proteins originated from sequences of msfGFP (# 91902, Addgene), mNeonGreen (#58179, Addgene), Twitch-3 (#49532, Addgene), Twitch-2B (#100040, Addgene), and mClover3 (#74252, Addgene), respectively. We added a nuclear-export sequence (MLQNELALKLAGLDINKTG)^10,56^ at the N-terminus to localize the sensor expression to the cytoplasm. The sequences of all sensors are in Supplementary Table 2. To express the fused proteins in HEK293 cells, we inserted the genes into a lentivirus backbone under the CamkIIα promoter (#48762, Addgene). To express the fused proteins in cultured neurons and in live mice, we inserted the genes into an adeno-associated virus backbone under the CamkIIα promoter (#26969, Addgene). To express the fused protein in bacteria, we inserted the genes into the pET-28b backbone (69865-3, Millipore Sigma) while fusing the sensors to a 6× His tag.

### One-photon and two-photon microscopy

We performed one-photon microscopy on all cultured cells using a commercial fluorescence microscope (E600FN, Nikon). We produced images and movies of the cells simultaneously in two fluorescence channels using a Dual-view adapter (DV2, Photometrics) and a sCMOS camera (OptiMOS, QImaging). When imaging our green-red fluorescent sensors, we illuminated samples using a cyan LED (M490L4, Thorlabs) with an intensity of 5 mW/mm^2^ at the sample plane. The excitation path contained a 480/30 nm filter (AT480/30x, Chroma) followed by a 505 nm long pass dichroic mirror (T505lpxr, Chroma). The emission path after the dichroic mirror contained a 565 nm long pass dichroic within the Dualview, followed by a 540/50 nm emission filter (HQ540/50 m, Chroma) or 620/60 nm emission filter (ET620/60 m, Chroma) matched to the green or red fluorescence of the sensors, respectively. When imaging the Twitch-3 or Twitch-2B sensors, we illuminated samples using a violet LED (M450LP, Thorlabs) with an intensity of 5 mW/mm^2^ at the sample plane. The excitation path contained a 436/20 nm excitation filter (ET436/20x, Chroma) followed by a 455 nm long pass dichroic mirror (T455lp, Chroma). The emission path after the dichroic mirror contained a 505 nm long pass dichroic mirror within the Dual-view, followed by a 480/40 nm emission filter (ET480/40 m, Chroma) or 540/50 nm emission filter (HQ540/50 m, Chroma) matched to the cyan or yellow fluorescence of the sensors, respectively.

We recorded the spectra of cells and purified protein solutions using a spectrometer (Flame UV-VIS, Ocean optics) in place of the camera with a 1 s integration time. We used a wide bandwidth white light LED (Thorlabs, MWWHLP1) as the excitation source. To record the full spectra of the green-red sensors, we used a 480/30 nm excitation filter (AT480/30x, Chroma), a 505 nm long pass dichroic mirror (T505lpxr, Chroma), and a 496 nm long-pass emission filter (FF01-496/LP, Semrock). To record the full spectra of the cyan-yellow sensors, we used a 436/20 nm excitation filter (ET436/20x, Chroma), a 455 nm long pass dichroic mirror (T455lp, Chroma), and a 458 nm long-pass emission filter (LP03-458RU, Semrock). To target the acceptor spectrum of green-red sensors, we used a 540/30 nm excitation filter (ET540/30 m, Chroma), a 565 nm long pass dichroic mirror (T565lpxr, Chroma), and a 620/60 nm emission filter (ET620/60 m, Chroma). To target the acceptor spectrum of cyan-yellow sensors, we used a 480/30 nm excitation filter (AT480/30x, Chroma), a 505 nm long pass dichroic mirror (T505lpxr, Chroma), and a 535/50 nm emission filter (ET535/50 m, Chroma).

We performed two-photon imaging of layer 2/3 and layer 5 neurons in the mouse visual cortex during awake behavior using a custom-built resonant two-photon microscope. The microscope employed a Chameleon Vision-S pulsed laser (Coherent) as the excitation source. The excitation wavelength was 930 nm for imaging Twitch-GR and 860 nm for imaging Twitch-2B. The excitation power was 20–50 mW for layer 2/3 imaging and 80–100 mW for layer 5 imaging. We used a 16×/0.8 NA objective (CFI75 LWD 16X W, Nikon) to focus the excitation light and collect the emission. We then directed the collected emission toward a 735 nm long-pass dichroic filter (FF735-Di02, Semrock) and through a 380–720 nm bandpass filter (FF01-750/SP, Semrock). When imaging Twitch-GR, we split the donor and acceptor emission using a 573 nm long-pass dichroic mirror (FF573-Di01, Semrock), and further filtered each channel using 525/50 nm and 650/100 nm bandpass filters (FF03-525-50, FF01-650-100, Semrock) for the green and red channels, respectively. When imaging Twitch-2B, we split the donor and acceptor emission using a 506 nm long-pass dichroic mirror (FF506-DI03, Semrock), and further filtered each channel using 480/40 nm and 535/50 nm bandpass filters (ET480/40 m, Chroma; FF01-535/50, Semrock) for the cyan and yellow channels, respectively. We used ScanImage 2017 (Vidrio Technologies) to scan both channels at a 44 Hz frame rate over a 512 × 512 pixel region; this region corresponded to a 100 µm × 100 µm to 250 µm × 250 µm field of view.

### HEK293 cell culture

We cultured HEK293T cells (Takara Biosciences) in Dulbecco Modified Eagle Medium (DMEM) (#10569010, Gibco) supplemented with 10% fetal bovine serum (FBS) (#16000044, Gibco) and 1% penicillin-streptomycin (#15070063, Gibco). One day after passaging the cells, we transfected the cells by mixing 1 μL of Lipofectamine 3000 (L3000015, Invitrogen) and 500 ng of DNA plasmid in optiMEM (#31985070, Gibco), and incubated the mixture with the cells for 5 h. We passaged the cells to coverslips one day after the transfection and imaged the cells after an additional day.

### Neuron culture and transfection

All animal handling and imaging procedures were performed according to protocols approved by the Duke Institutional Animal Care and Use Committee (IACUC). We obtained rat hippocampal neurons from postnatal day 0 Sprague-Dawley pups (Charles River Labs) by hippocampal dissection, papain-based dissociation, and plating on Matrigel-coated glass coverslips^57^. We cultured neurons in Neurobasal Media A (#10888022, Gibco) supplemented with GlutaMAX (A1286001, Gibco) and B-27 Supplement (#17504044, Gibco). Three to five days after plating, we transfected the neurons using calcium phosphate; we incubated a mixture of the calcium phosphate buffer and 1 μg of DNA plasmid per well for 40–60 min. We imaged the neurons an additional three to five days after transfection to allow for robust sensor expression.

### HEK cell and neuron manipulations

We placed coverslips with cultured neurons in a perfusion chamber in which the extracellular media consisted of 150 mM NaCl, 4 mM KCl, 10 mM glucose, 10 mM HEPES, 2 mM CaCl_2_, and 2 mM MgCl_2_. We pulled glass pipettes with resistances of 3–5 MΩ, and filled the pipettes with intracellular solution consisting of 129 mM K-gluconate, 10 mM KCl, 10 mM HEPES, and 4 mM Na_2_ATP. To characterize the Ca^2+^ response of sensors, we treated the cultured HEK293 cells with the same extracellular solution that included an additional 10 μM of ionomycin and 5 mM of Ca^2+^ to greatly increase the cytosolic Ca^2+^ concentration. We named the conditions before and after the ionomycin treatment as the Ca^2+^-resting condition and Ca^2+^-saturated condition, respectively.

We loose-patched cultured neurons using an Axon Digidata 1550 A digitizer (Axon Instruments), a Multiclamp 700 A amplifier (Axon Instruments), and pClamp (version 7.1) software at room temperature. We touched the pipettes on neurons and formed partial seals with resistances of 30–50 MΩ. We electrically stimulated neurons using 2 ms pulses at intervals of 500 ms and current amplitudes ranging from 300 to 1500 pA. We manually removed stimulus artifacts from the electrophysiology traces. Dual-view fluorescent imaging simultaneously recorded the intensity of patched neurons in the donor and acceptor channels at 10 Hz.

### FRET efficiency, quantum yield, and sensor brightness calculations

We quantified the FRET efficiency and molecular brightness of sensors expressed in individual HEK293 cells under all manipulations by measuring both the total FRET spectrum (*F*_total_(*λ*)) and the acceptor-only spectrum (*F*_AO_(*λ*)). We obtained *F*_total_ by exciting the sensors with wavelengths targeted to the donor excitation peak. *F*_total_ included emission from both the donor component of our sensors, with amplitude *F*_D_, and the acceptor component of our sensor, with amplitude *F*_A_:

1 $${F}_{{{{{{\rm{total}}}}}}}(\lambda )={F}_{{{{{{\rm{D}}}}}}}D(\lambda )+{F}_{{{{{{\rm{A}}}}}}}A(\lambda ),$$

where *D*(*λ*) and *A*(*λ*) were respectively the spectra of the donor fluorescent protein and acceptor fluorescent protein normalized by the wavelength-integrals of their respective spectra. To extract the amplitudes of *F*_D_ and *F*_A_, we fitted *F*_total_ to a linear combination of the reported spectra of the donor and acceptor fluorescent proteins from FPbase (https://www.fpbase.org). We fitted the total spectrum of Twitch-3 to the spectra of ECFP and mCitrine, and the total spectrum of Twitch-2B to the spectra of mCerulean3 and mVenus. We fitted all total spectra of green-red sensors to the spectra of mScarlet and their respective donor fluorescent proteins: the donor fluorescent protein spectra for Twitch-GR, Twitch-NR, Twitch-CiR, Twitch-ClR, and Twitch-VR were the spectra for superfolder GFP, mNeon, mCitrine, mClover, and mVenus, respectively.

The donor emission arose from the fraction of excited donor proteins that did not transfer energy via FRET to the acceptors; the acceptor emission arose from the combination of acceptor excitation at the donor excitation wavelength and FRET transfer excitation:

2 $${F}_{{{{{{\rm{D}}}}}}}=c{I}_{{{{{{\rm{D}}}}}}}{\varepsilon }_{{{{{{\rm{D}}}}}}}{{QY}}_{{{{{{\rm{D}}}}}}}\left(1-E\right)$$

3 $${F}_{{{{{{\rm{A}}}}}}}={{cEI}}_{{{{{{\rm{D}}}}}}}{\varepsilon }_{{{{{{\rm{D}}}}}}}{{QY}}_{{{{{{\rm{A}}}}}}}+c{I}_{{{{{{\rm{D}}}}}}}{\varepsilon }_{{{{{{\rm{A}}}}}}}{{QY}}_{{{{{{\rm{A}}}}}}},$$

where *ε*_D_ and *ε*_A_ were, respectively, the average extinction coefficients of the donor and acceptor fluorescent proteins within the excitation band of the donor excitation filter, *QY*_D_ and *QY*_A_ were respectively the quantum yields of the donor and acceptor fluorescent proteins, *E* was the FRET efficiency between the donor and acceptor, *c* was the number of sensor molecules, and *I*_D_ was the excitation intensity through the filter targeted to the excitation peak of the donor.

We experimentally obtained *F*_AO_ by exciting the sensors with wavelengths targeted to the acceptor excitation peak. We then found the acceptor amplitude $({F}_{{{{{{\rm{A}}}}}}}^{{\prime} })$ by fitting *F*_AO_(*λ*) to the acceptor fluorescent protein spectrum reported on the FPbase. The resulting intensity arose from only excitation of the acceptor:

4 $${F}_{{{{{{\rm{A}}}}}}}^{{\prime} }={{cI}}_{{{{{{\rm{A}}}}}}}{\varepsilon }_{{{{{{\rm{A}}}}}}}^{{\prime} }{{QY}}_{{{{{{\rm{A}}}}}}},$$

where ${\varepsilon }_{{{{{{\rm{A}}}}}}}^{{\prime}}$ was the average extinction coefficient of the acceptor within the excitation band of the acceptor excitation filter, and *I*_A_ was the intensity of the light excitation through the excitation filter. Assuming that the extinction spectra and protein numbers were constant between the two spectra, and that the truncations or circular permutations only changed the quantum yields of the truncated or circularly permuted fluorescent proteins, respectively, we solved for the FRET efficiency and the ratio between the quantum yields of the two sensor fluorescent proteins from the system of Eqs. (2)-(4), as the following:

5 $$E=\frac{{{F}_{{{{{{\rm{A}}}}}}}I}_{{{{{{\rm{A}}}}}}}{\varepsilon }_{{{{{{\rm{A}}}}}}}^{{\prime} }-{F}_{{{{{{\rm{A}}}}}}}^{{\prime} }{I}_{{{{{{\rm{D}}}}}}}{\varepsilon }_{{{{{{\rm{A}}}}}}}}{{F}_{{{{{{\rm{A}}}}}}}^{{\prime} }{I}_{{{{{{\rm{D}}}}}}}{\varepsilon }_{D}}$$

6 $$\frac{{{QY}}_{{{{{{\rm{A}}}}}}}}{{{QY}}_{{{{{{\rm{D}}}}}}}}=\frac{{F}_{{{{{{\rm{A}}}}}}}^{{\prime} }{I}_{{{{{{\rm{D}}}}}}}{\varepsilon }_{{{{{{\rm{D}}}}}}}}{{F}_{{{{{{\rm{D}}}}}}}{I}_{{{{{{\rm{A}}}}}}}{\varepsilon }_{{{{{{\rm{A}}}}}}}^{{\prime} }}\times (1-E)$$

We used the ratio between the quantum yields of the two sensor fluorescent proteins to quantify the donor quantum yield when the acceptor quantum yield was known, and the acceptor quantum yield when the donor quantum yield was known. We then defined the molecular brightness of the donor and acceptor channels of sensor, *B*_D_ and *B*_A_, as the following equations:

7 $${B}_{{{{{{\rm{D}}}}}}}={\varepsilon }_{{{{{{\rm{D}}}}}},{{{{{\rm{peak}}}}}}}Q{Y}_{{{{{{\rm{D}}}}}}}\left(1-E\right)$$

8 $${B}_{{{{{{\rm{A}}}}}}}={\varepsilon }_{{{{{{\rm{D}}}}}},{{{{{\rm{peak}}}}}}}Q{Y}_{{{{{{\rm{A}}}}}}}E+{\varepsilon }_{{{{{{\rm{A}}}}}},{{{{{\rm{peak}}}}}}}Q{Y}_{{{{{{\rm{A}}}}}}},$$

where ${\varepsilon }_{{{{{\rm{D}}}}},{{{{\rm{peak}}}}}}^{\prime}$ was the peak donor extinction coefficient, and ${\varepsilon }_{{{{{{\rm{A}}}}}},{{{{{\rm{peak}}}}}}}^{\prime}$ was the acceptor extinction coefficient at the peak wavelength of the donor extinction spectrum. We calculated the total molecular brightness *B*_total_ as:

9 $${B}_{{{{{{\rm{total}}}}}}}=\sqrt{{B}_{{{{{{\rm{D}}}}}}}^{2}+{B}_{{{{{{\rm{A}}}}}}}^{2}}.$$

Because SNR from individual ratiometric channels add in quadrature under shot-noise limited experiments to produce a total SNR^42^, our total brightness measure was representative of the expected baseline noise for the total SNR.

We used dual-view imaging to calculate cellular brightness. We defined regions of interest (ROIs) for both the donor image and the acceptor image. The integrated cellular intensities from the donor ROI (*F*_c,D_) and acceptor ROI (*F*_c,A_) helped calculate the total cellular brightness *F*_c,total_:

10 $${F}_{{{{{{\rm{c}}}}}},{{{{{\rm{total}}}}}}}=\sqrt{{F}_{{{{{{\rm{c}}}}}},{{{{{\rm{D}}}}}}}^{2}+{F}_{{{{{{\rm{c}}}}}},{{{{{\rm{A}}}}}}}^{2}}$$

### Characterization of sensor response and SNR

We defined multiple measures of shot-noise limited SNR for purified protein-based measurements and cell-based measures. These measures estimated the noise based on intensity measurements of the sensor, which is the theoretical shot-noise limit for fluorescent sensors. For measurements on individual fluorescence channels, the shot-noise-limited SNR was the following:

11 $${{{{{\rm{SNR}}}}}}\left(t\right)=\frac{F\left(t\right)-\bar{F}}{\bar{F}}\times \sqrt{\bar{F}},$$

where *F*(*t*) was the fluorescence trace of that channel and $\bar{F}$ was the fluorescence of that channel in the resting state. For experiments in HEK cells that went from the Ca^2+^-resting state to the Ca^2+^-saturated state, *F*(*t*) was the steady-state fluorescence in the Ca^2+^-saturated state. For experiments on purified proteins that went from the zero Ca^2+^ buffer to high Ca^2+^ buffer, *F*(*t*) was the steady-state fluorescence in the high Ca^2+^ buffer state. For experiments where the fluorescence reported neuronal action potentials, we reported the peak of SNR(*t*) as the SNR.

For metrics based on two fluorescence channels, we defined a ratiometric measure, *R*, that divided the acceptor fluorescence by the donor fluorescence:

12 $$R=\frac{{F}_{{{{{{\rm{A}}}}}}}}{{F}_{{{{{{\rm{D}}}}}}}}\;{{{{{\rm{or}}}}}}\; R=\frac{{F}_{{{{{{\rm{c}}}}}},{{{{{\rm{A}}}}}}}}{{F}_{{{{{{\rm{c}}}}}},{{{{{\rm{D}}}}}}}}{{{{{\rm{;}}}}}}$$

the former equation employed the respective fitted acceptor and donor fluorescence amplitudes from a spectral measurement, while the latter equation employed the respective spatially integrated acceptor and donor emissions from intensity measurements imaged in independently filtered channels. We defined the relative change in the ratiometric measure for each *R* measurement as the following:

13 $$\frac{\Delta R}{{R}_{0}}=\frac{R-{R}_{0}}{{R}_{0}},$$

where *R*_0_ is the baseline ratiometric measure. We further defined total SNR and ratiometric SNR for metrics based on measurements of multiple fluorescence channels.

*Total SNR*—after we found *F*_c_,_D_ and *F*_c_,_A_ through either linear fitting or integration, we defined the SNR of the donor channel (SNR_D_) and acceptor channel (SNR_A_) as:

14 $${{{{{\rm{SN}}}}}}{{{{{{\rm{R}}}}}}}_{{{{{{\rm{D}}}}}}}=\frac{{F}_{{{{{{\rm{c}}}}}},{{{{{\rm{D}}}}}}}-{F}_{{{{{{\rm{c}}}}}},{{{{{\rm{D}}}}}}0}}{{F}_{{{{{{\rm{c}}}}}},{{{{{\rm{D}}}}}}0}}\sqrt{{F}_{{{{{{\rm{c}}}}}},{{{{{\rm{D}}}}}}0}}$$

15 $${{{{{\rm{SN}}}}}}{{{{{{\rm{R}}}}}}}_{{{{{{\rm{A}}}}}}}=\frac{{F}_{{{{{{\rm{c}}}}}},{{{{{\rm{A}}}}}}}-{F}_{{{{{{\rm{c}}}}}},{{{{{\rm{A}}}}}}0}}{{F}_{{{{{{\rm{c}}}}}},{{{{{\rm{A}}}}}}0}}\sqrt{{F}_{{{{{{\rm{c}}}}}},{{{{{\rm{A}}}}}}0}},$$

where *F*_c,D0_ and *F*_c,A0_ were, respectively, the imaged donor and acceptor channel fluorescence intensities of the sensor in the resting state. The total SNR is the quadratic combination of the individual channel SNRs:

16 $${{{{{\rm{SN}}}}}}{{{{{{\rm{R}}}}}}}_{{{{{{\rm{total}}}}}}}=\sqrt{{{{{{{{\rm{SNR}}}}}}}_{{{{{{\rm{D}}}}}}}}^{2}+{{{{{{{\rm{SNR}}}}}}}_{{{{{{\rm{A}}}}}}}}^{2}}.$$

This metric optimally used shot-noise limited information from both channels^42^.

*Ratiometric SNR*—we also estimated the shot-noise limited SNR of the ratiometric measure *R*. Because our sensors were bright and we obtained more than 10 photons per measurement, we estimated the shot-noise distribution of each fluorescence channel with emission amplitude *F* as a Gaussian random variable with mean *F* and standard deviation $\sqrt{F}$. We then calculated *σ*_Ratio,_ the standard deviation of the ratiometric measurement as the standard deviation of the acceptor-to-donor ratio;^42^ this value represents the shot-noise of *R*. We calculated the ratiometric SNR (SNR_r_) as the following:

17 $${{{{{{\rm{SNR}}}}}}}_{{{{{{\rm{r}}}}}}}=\frac{{R}_{{{{{{\rm{peak}}}}}}}-{R}_{0}}{{\sigma }_{{{{{{\rm{Ratio}}}}}}}},$$

where *R*_peak_ was the peak response of the calcium transient measured by *R*, and *R*_0_ was the baseline response measured by *R*.

*SNR calculation for two-photon imaging*—we manually defined ROIs of individual neuron somas and defined 5 pixel thick round-shape regions outside the ROIs as the region for calculating the neuropil background. We defined the unmixed fluorescence trace of individual neurons as

18 $$F=\frac{\left({F}_{{{{{{\rm{soma}}}}}}}-0.7{F}_{{{{{{\rm{neuropil}}}}}}}\right)N}{G},$$

where *F*_soma_ was the average ScanImage count from the soma ROI, *F*_neuropil_ was the average of ScanImage counts from the neuropil background region, *N* was the pixel number of the soma ROI, and *G* was the gain of the PMT. The 0.7 fraction of the neuropil fluorescence was previously used to remove background fluorescence in two-photon imaging experiments^6^. We then calculated the ratiometric SNR trace (SNR_r_) from the donor and acceptor channel traces.

Because we imaged different layers of the brain expressing Twitch-GR and Twitch-2B with different excitation powers, we normalized the fluorescence of the neurons using the following conversion:

19 $${{{{{{\rm{SNR}}}}}}}_{{{{{{\rm{Norm}}}}}}}={{{{{\rm{p}}}}}}{{{{{{\rm{SNR}}}}}}}_{{{{{{\rm{r}}}}}}}\frac{50\,{{{{{\rm{mW}}}}}}}{P},$$

where pSNR_r_ was the peak SNR of the average ratiometric response from individual neurons after visual stimulus and *P* was the excitation power. SNR_Norm_ thus represented the shot-noise limited ratiometric SNR if the two-photon imaging experiment were conducted with 50 mW excitation power.

### Expression and purification of proteins

We transformed the bacterial expression vector containing our sensors in *E. coli* BL21 (DE3) cells (EC0114, ThermoFisher Scientific). We cultured the transformed cells in fresh LB medium at 37 °C overnight and transferred 100 μL of this solution into 100 mL of fresh LB medium. We induced expression of the sensors in this bath by adding 0.5 mM IPTG into the media. We then grew the bacteria for 2 h at 37 °C to express the protein, and shook an additional 4 h at 20 °C to improve protein maturation. We collected bacteria pellets by using centrifugation at 10,000 *g*. We then used a HOOK 6× His protein Spin Purification Kit (#786‐628, G-Biosciences) to extract the sensor proteins by chelating the His6 tag of the sensors to Nickel magnetic beads. We dialyzed the protein elution buffer overnight in PBS pH 7.4 using a 4 kDa membrane tubing (#786-616, G-Biosciences), and stored the resulting protein solution at 4 °C for 1–3 days or at −80 °C for longer periods.

### Characterization of sensor Ca^2+^ affinity

We prepared Ca^2+^ baths of specific concentrations by using a calcium calibration buffer kit (59100, Biotium). The kit included two stock solutions: (1) a zero calcium buffer containing 10 mM K_2_EGTA, 100 mM KCl, and 10 mM MOPS; (2) a high calcium buffer containing 10 mM CaEGTA, 100 mM KCl, and 10 mM MOPS. We mixed purified sensor protein solution into each of these two buffers at 5 μM. We then mixed appropriate amounts of the two solutions together to titrate the Ca^2+^ concentration between 0 to 39.8 μM. We recorded the spectrum of each solution over a 1 s integration time. From these spectra, we again quantified the relative change in the ratiometric measure (Δ*R*/*R*_0_), the FRET efficiency, and the quantum yield as in the previous sections. We then normalized the Δ*R*/*R*_0_ response to the response at 39.8 μM Ca^2+^. Finally, we found the affinity constant *K*_*d*_ by fitting the normalized Ca^2+^ response curve to a first-order Hill model using linear regression.

### Photobleaching measurement

We imaged HEK293 cells expressing sensors using the same dual-view microscope and filter sets as the microscope used for imaging all cells. We imaged using an excitation intensity of 5 mW/mm^2^, and collected fluorescence for 500 s at 0.5 s intervals. We registered the donor and acceptor channel images over this duration. We again manually defined ROIs corresponding to individual cells and calculated the fluorescence traces. We calculated the photobleaching time constants by fitting the fluorescence time series to a single exponential curve.

### Sensor kinetics calculation

We fitted the fluorescence traces from our experiments to the following equation:

20 $$F(t)={{{{{{\rm{e}}}}}}}^{-\frac{t}{{\tau }_{{{{{{\rm{off}}}}}}}}}\left(1-{{{{{{\rm{e}}}}}}}^{-\frac{t}{{\tau }_{{{{{{\rm{on}}}}}}}}}\right),$$

where *F*(*t*) was the fluorescence trace of sensor, *t* was time, *τ*_on_ was the rise time constant, and *τ*_off_ was the decay time constant. This equation captures first-order rise and fall dynamics that occur simultaneously with different kinetics.

### Mouse surgery and preparation for in vivo imaging

During the injection and implant surgeries, we anesthetized mice using isoflurane (5% for induction, 1.0–1.5% during surgery). We injected 600 nL of 3 × 10^12^ vg/mL titer adeno-associated virus (AAV-CamkII-Twitch-GR, AAV-CamkII-Twitch-2B, and AAV-CamkII-GCaMP6s) into the right visual cortex of 8 weeks old male C57BL/J6 mice (Jackson Laboratory). The injecting location was 3.5 mm posterior to bregma, 2.5 mm lateral from the midline, and 0.45 mm deep. Two weeks after the injection, we placed a 3.2 mm diameter circular craniotomy above the injection site and then covered the craniotomy with a cannula sealed by a 3 mm diameter round glass coverslip (#640720, Warner Instruments) on one end. We glued the cannula to the skull using UV glue (NOA61; Norland). We then fixed the outlying scalp to the skull using Metabond quick adhesive cement (S380, Parkell). We finally secured the entire implant and a custom titanium head post to the skull using dental cement (#51459, Stoelting). We imaged mice 2–6 weeks after the implant surgery.

### Mouse visual stimuli for in vivo imaging

We showed visual stimuli with a liquid crystal display centered at 25 cm in front of the mice’s left eye. The monitor subtended a ± 38° horizontal angle and ±31° vertical angle around the mouse’s eye. Visual stimuli were drifting gratings generated using Psychtoolbox-3 in MATLAB (http://psychtoolbox.org). We presented drifting sinusoidal grating stimuli (0.05 cycles per degree spatial frequency, 1 Hz temporal frequency) at 8 directions (4 cardinal, 4 intercardinal). We presented each direction of stimulus for 2 s, followed by a 6 s blank stimulus period (uniform gray at the mean luminance of the monitor). We synchronized the visual stimuli to individual two-photon frames using the frame-start pulses provided by ScanImage.

### Statistics and reproducibility

We listed the tests and sample numbers alongside all statistical tests.

### Reporting summary

Further information on research design is available in the Nature Research Reporting Summary linked to this article.

## Supplementary information

Supplementary Information

Reporting Summary

## Data Availability

Twitch-GR (#173044) and Twitch-NR (#173043) are available on Addgene. Source data associated with reproducing the main figures can be found at 10.6084/m9.figshare.14842467^58^. Other datasets generated during and/or analyzed during the current study are available from the corresponding author on reasonable request.

## Code availability

Code for labeling neurons can be found at https://github.com/soltanianzadeh/STNeuroNet/ arising from previous work^59^. Other code used for analysis are available from the corresponding author on reasonable request.
