# Supplementary material for: Rational engineering of ratiometric calcium sensors with bright green and red fluorescent proteins
Source: Commun Biol. 2021 Jul 29;4:924. doi: 10.1038/s42003-021-02452-z (PMC8322158; doi:10.1038/s42003-021-02452-z)
Supplement: Supplementary file 1 — Supplementary Information [file 42003_2021_2452_MOESM1_ESM.pdf]

Supplementary Information

Rational Engineering of Ratiometric Calcium Sensors with Bright Green and Red Fluorescent Proteins for Neural Calcium Imaging

Diming Zhang, Emily Redington, Yiyang Gong

emails: diming.zhang@duke.edu, yiyang.gong@duke.edu

Affiliations: Department of Biomedical Engineering, Duke University, Durham, NC 27708, USA

Supplementary Figure 1 – HEK293 cell spectra characterized sensor folding and FRET efficiency.

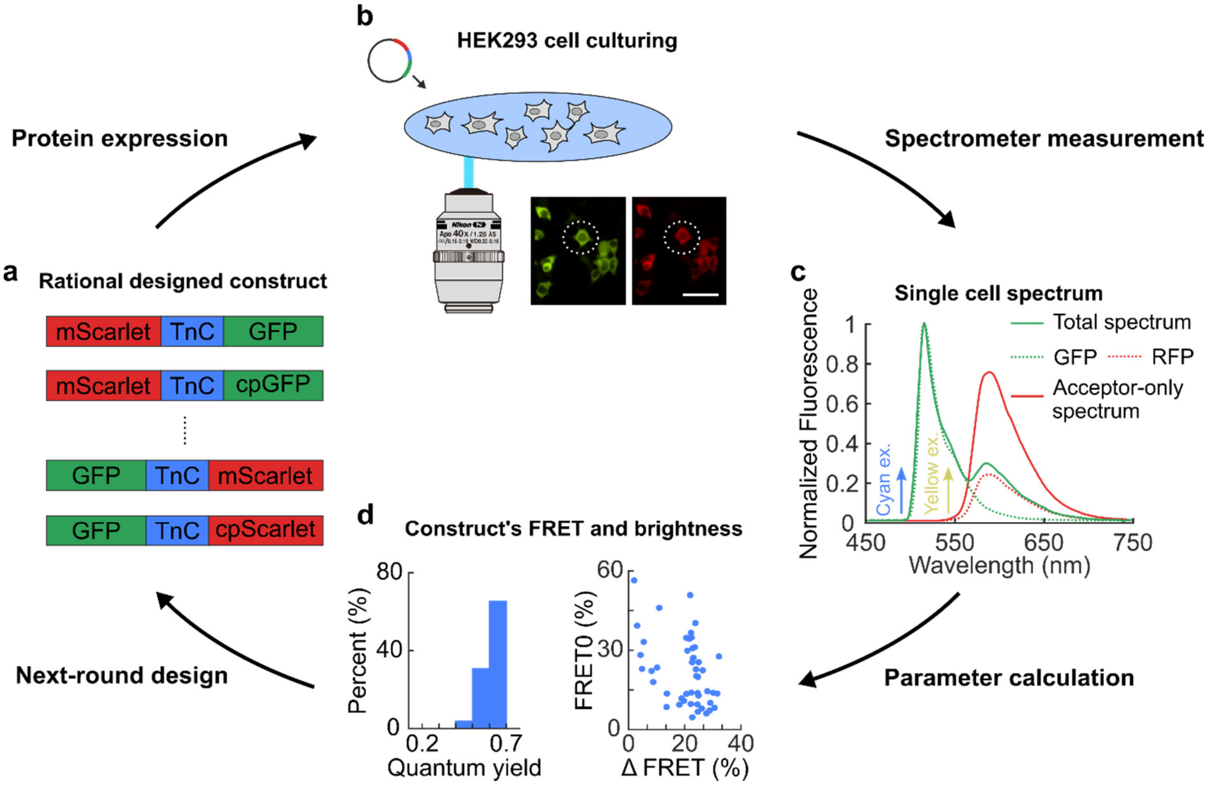

(a) Sensor candidates linked a red fluorescent protein (mScarlet) and a green-fluorescent protein (GFP) analog to the calcium sensing domain. The cpmScarlet was a circular permutation of mScarlet about the 169th residue. **(b)** Images of sensor expression in HEK293 cells. The scale bar is 30 μm. **(c)** Spectra of single cells expressing green-red sensor candidates when illuminated by cyan or yellow excitation light are shown by solid lines. Spectral fits shown by the dashed lines break the "total spectrum" spectrum down to its component GFP and RFP spectra. **(d)** The spectra of the sensors quantified the quantum yield of the fluorescent proteins and the FRET efficiency of the sensors. *Left*: A histogram of quantum yield for the red fluorescent emitter in a set of cells. *Right*: a scatter plot of baseline FRET efficiency (FRET0) vs. change in FRET efficiency between the baseline state and high calcium state (ΔFRET). The quantum yield served as a proxy for the folding quality of the fluorescent proteins, while the FRET efficiency served as a proxy for the Ca^{2+} sensitivity of the sensors.

Supplementary Figure 2 – Various arrangements of the fluorescent proteins on the calcium sensing domain produced different calcium sensitivities.

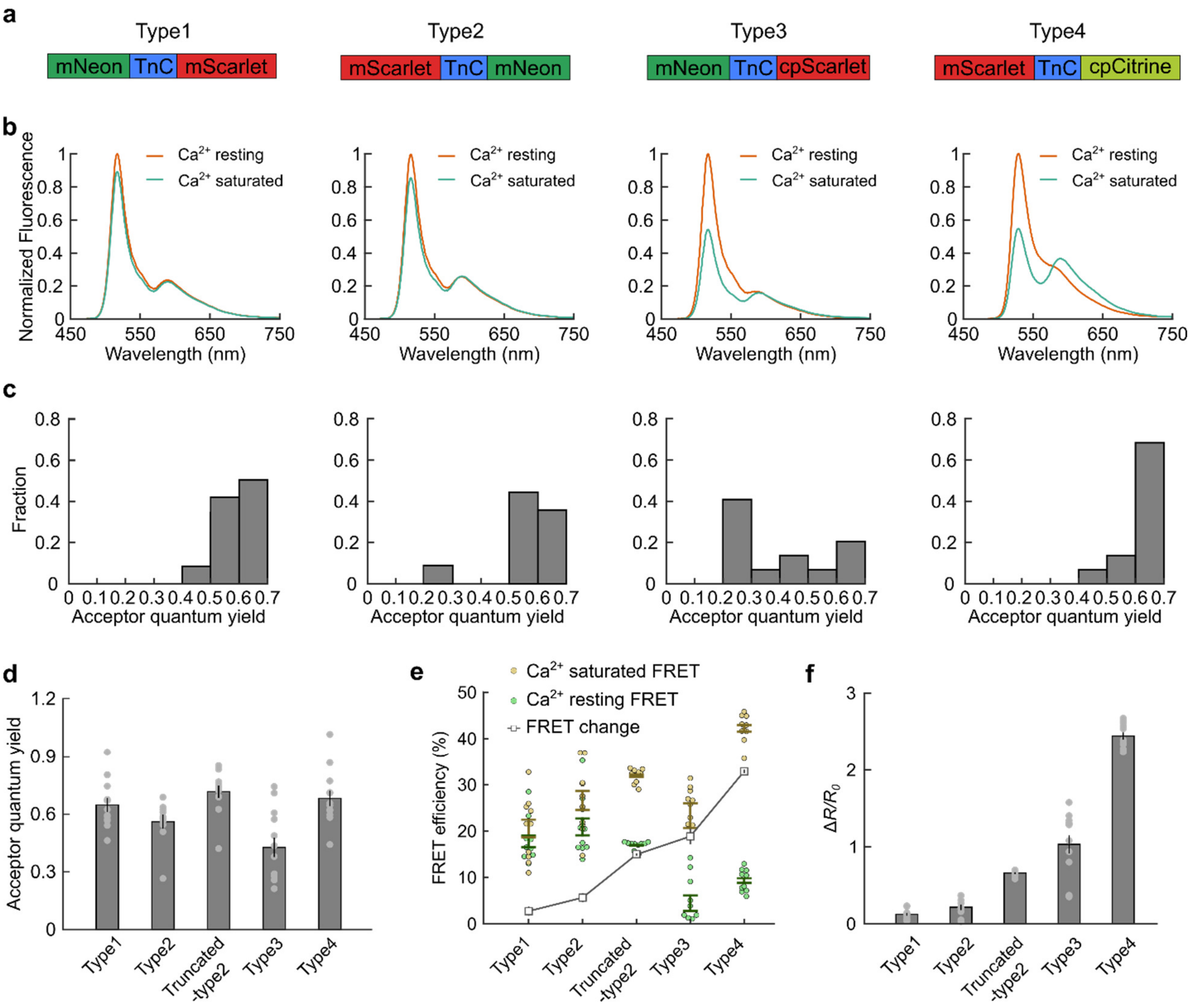

(a) Four configurations linked various fluorescent proteins at different positions on the calcium sensing domain. The cpScarlet was a circular permutation of mScarlet about the 169th residue**.** **(b)** The spectra of each sensor configuration expressed in HEK293 cells under Ca^{2+}-resting and Ca^{2+}-saturated conditions. **(c)** The distribution of the sensors' acceptor quantum yield. **(d)** The acceptor quantum yield of the four sensor configurations. Gray dots indicate individual data points. **(e)** The FRET efficiency in the Ca^{2+}-resting and Ca^{2+}-saturated states of the four sensor configurations. **(f)** The change in the ratiometric measure between the Ca^{2+}-resting and Ca^{2+}-saturated states. The Truncated-type2 sensor was a version of the Type2 sensor that truncated 9 amino acids from the C-terminus of mScarlet and 12 amino acids from the N-terminus of mNeon. The Truncated-type2 sensor increased the FRET efficiency change and $\Delta R/R_0$ by ~2.5 times

compared to the Type2 sensor, but still had worse metrics than the Type3 and Type4 sensors, which were sensors using one circularly permuted fluorescent protein. Gray dots indicate individual data points. All error bars are s.e.m, $n \geq 10$ cells.

Supplementary Figure 3 – Truncation of amino acids on the C-terminus of mScarlet extinguished the red fluorescence of the mScarlet-TnC-cpCitrine sensors.

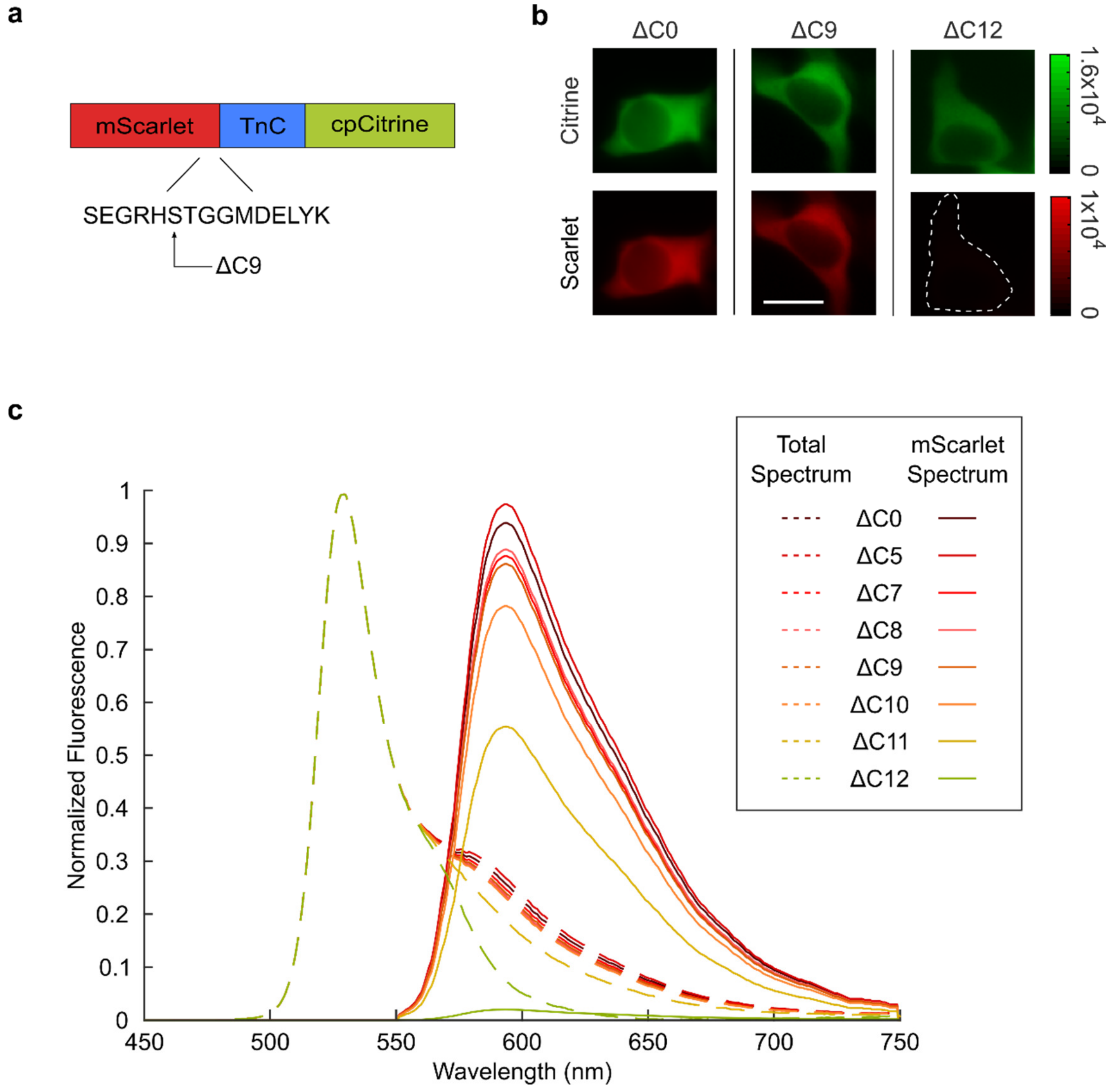

(a) The mScarlet-TnC-cpCitrine sensor design, highlighting the amino acid sequence that links the C-terminus of mScarlet to the N-terminus of the calcium sensing domain. **(b)** Dual-view images of HEK293 cells expressing mScarlet-TnC-cpCitrine sensors with different mScarlet C-terminus truncations. The dashed line is the outline of the cell in the red fluorescence channel. The 12 amino acid truncation eliminated red fluorescence. The scale bar is 20 μm. **(c)** The average spectra of the individual fluorescent protein components of the sensors as the truncation of the mScarlet C-terminus increased from 0 to 12 amino acids. ΔC0 corresponded with truncating to the 232th amino acid of mScarlet. We acquired the fluorescence spectrum in response to 488 nm excitation ("Total Spectrum", *dashed lines*). We then extracted the normalized mScarlet

fluorescence spectrum (mScarlet Spectrum, *solid lines*) from the Total Spectrum. Truncations of up to 9 amino acids only slightly decreased the emission intensity of the mScarlet component. Spectra are averaged over n > 10 HEK293 cells for each sensor.

Supplementary Figure 4 – Circular permutations of GFP-analogs retained their quantum yields relative to their native forms and increased the FRET efficiency change between the Ca^{2+}-resting and Ca^{2+}-saturated states.

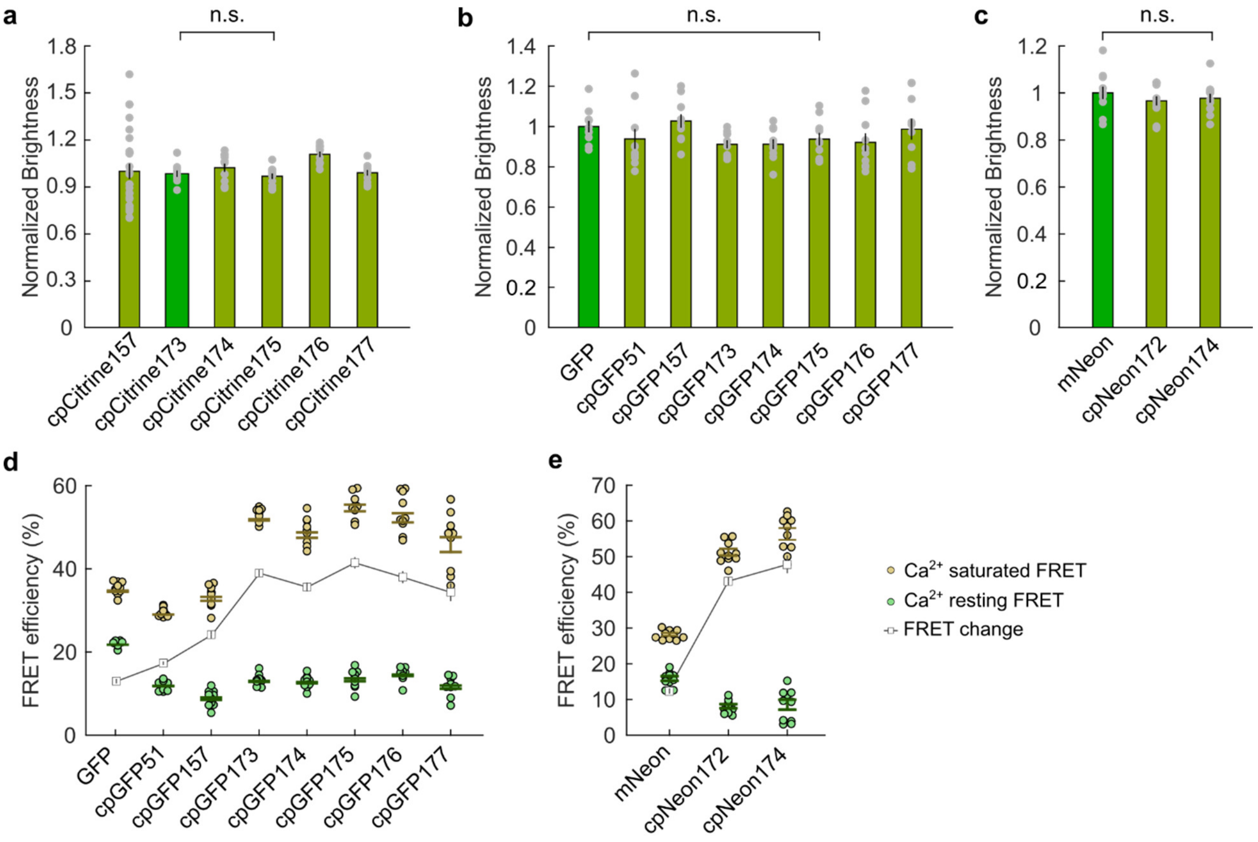

(a) The normalized brightness of various mCitrine circular permutations normalized to the brightness of cpCitrine173, the circularly permuted variant of mCitrine in the Twitch3 sensor. cpCitrine175, the donor of our best mCitrine-mScarlet Twitch sensor, had a brightness that was not significantly different than the brightness of the existing cpCitrine173 (p = 0.75, two-sided Wilcoxon rank-sum test, $n \geq 10$ cells for each variant). Gray dots indicate individual data points. **(b)** The brightnesses of different circularly permuted sfGFPs were similar to that of native sfGFP. cpGFP175, the donor within our best GFP-mScarlet GECI, had a brightness that was not significantly different than the brightness of native sfGFP (p = 0.62, two-sided Wilcoxon rank-sum test, $n > 10$ cells). Gray dots indicate individual data points. **(c)** The brightnesses of various mNeon circular permutations were similar to that of mNeon. cpNeon174, the donor within our best mNeon-mScarlet GECI, had a brightness that was not significantly different than the brightness of native mNeon (p = 0.56, two-sided Wilcoxon rank-sum test, $n > 10$ cells). Gray dots indicate individual data points. The FRET efficiencies of **(d)** sensors employing the various cpGFP circular permutations and **(e)** sensors employing the various cpNeon circular permutations in the Ca^{2+}-

resting or Ca^{2+}-saturated states, along with the change in FRET efficiency between the two states. All error bars are s.e.m., with $n \geq 10$ cells for each sensor. n.s. – not significant.

Supplementary Figure 5 – The spectra of sensors and fluorescent protein components in the Ca^{2+}-resting and Ca^{2+}-saturated states.

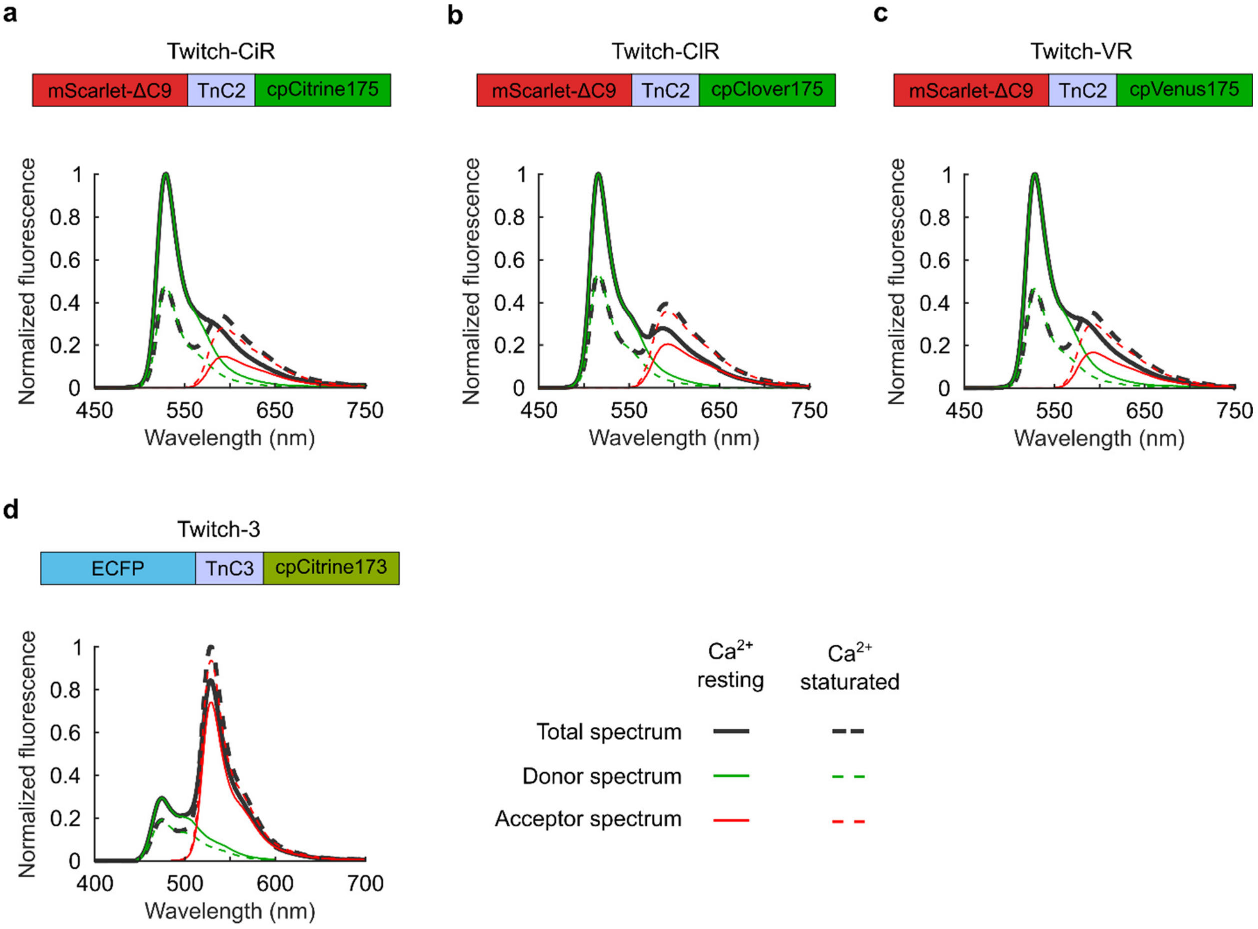

We obtained the Total spectra of **(a)** Twitch-CiR, **(b)** Twitch-ClR, **(c)** Twitch-VR, and **(d)** Twitch-3 when illuminating with wavelengths targeted to the donor. The “Total spectrum” was the full spectrum, while the “Donor” and “Acceptor” spectra were the fits to the spectra of the fluorescent protein components. All sensors showed a decrease in donor fluorescence and an increase in acceptor fluorescence as the Ca^{2+} concentration increased.

Supplementary Figure 6 – The Ca^{2+} affinity of TnC2 sensor variants were higher than the Ca^{2+} affinity of TnC3 sensor variants when measured by Ca^{2+} titration.

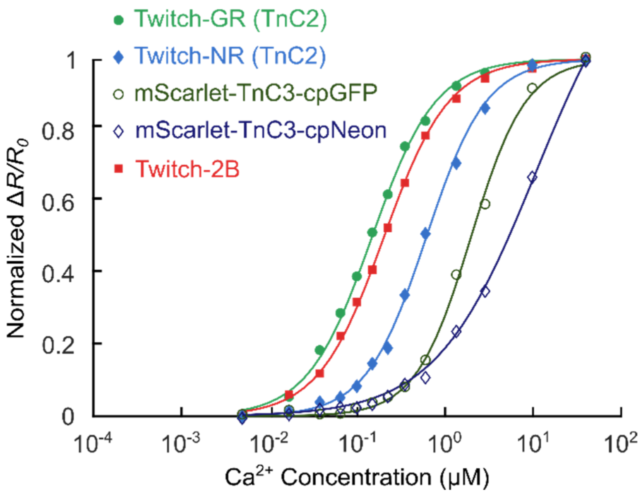

We measured the Ca^{2+} titration curves for multiple green-red sensors and Twitch-2B. Twitch-GR and Twitch-NR employed TnC2 as the calcium sensing domain. mScarlet-TnC3-cpGFP and mScarlet-TnC3-cpNeon employed TnC3 as the calcium sensing domain, and used the same fluorescent pairs as Twitch-GR and Twitch-NR, respectively. The solid lines are the fits between the experimental data and a first-order Hill model obtained by linear regression.

Supplementary Figure 7 – Photobleaching of GFP-RFP sensors occurred more slowly than photobleaching of CFP-YFP sensors.

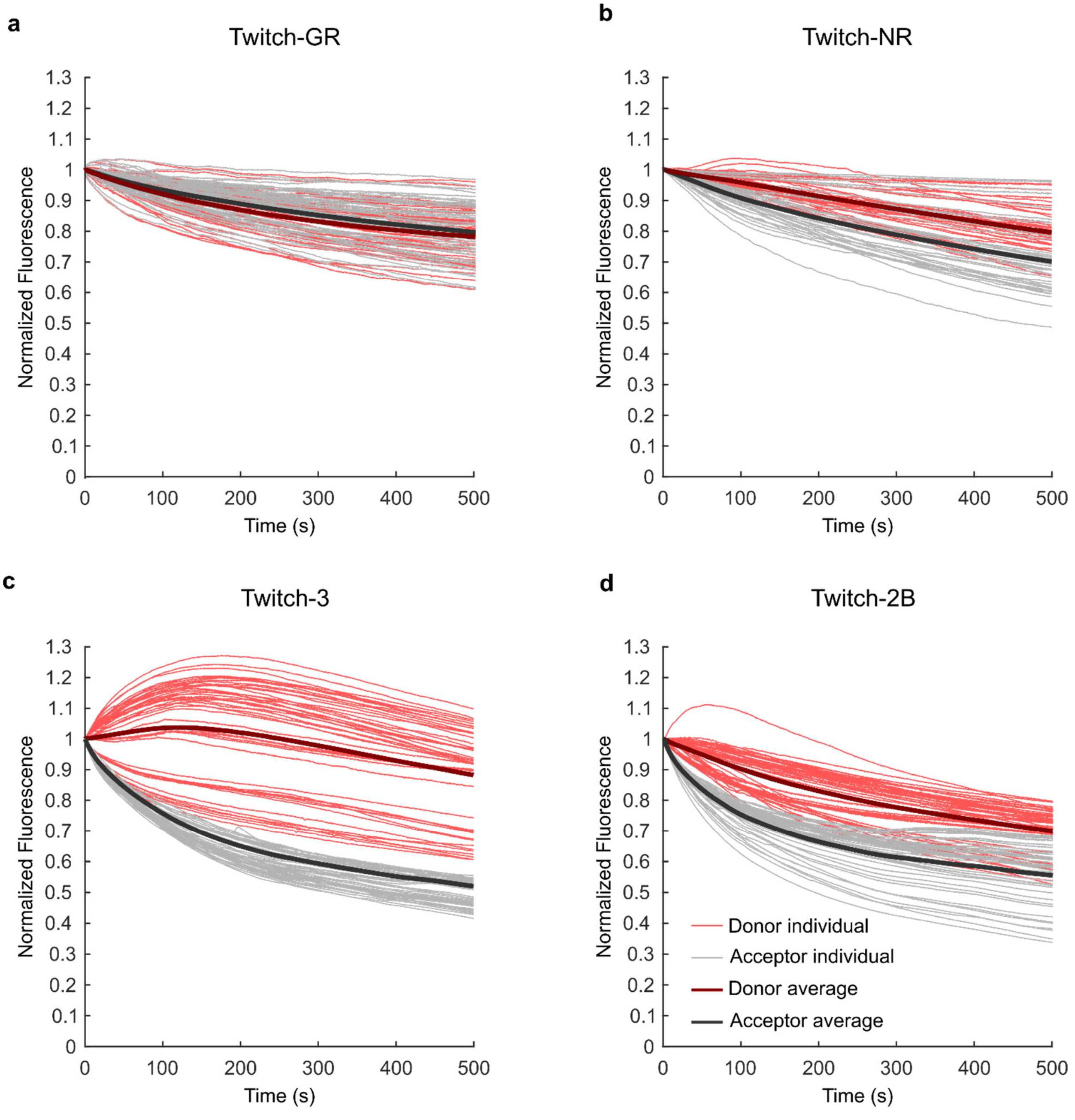

Photobleaching curves from individual cells expressing **(a)** Twitch-GR, **(b)** Twitch-NR, **(c)** Twitch-3, and **(d)** Twitch-2B. The thick, darker lines indicate the average of photobleaching curves from individual cells (*n* ≥ 30 cells). Most cells expressing Twitch-GR, Twitch-NR, and Twitch-2B followed an exponential decay. Twitch3 displayed transient increases in the donor emission, likely due to photoswitching of the CFP acceptor. The excitation power of both experiments was 5 mW/mm^2.

Supplementary Figure 8 – The SNR of Twitch-GR's donor and acceptor responses were larger than SNR of Twitch-2B's responses.

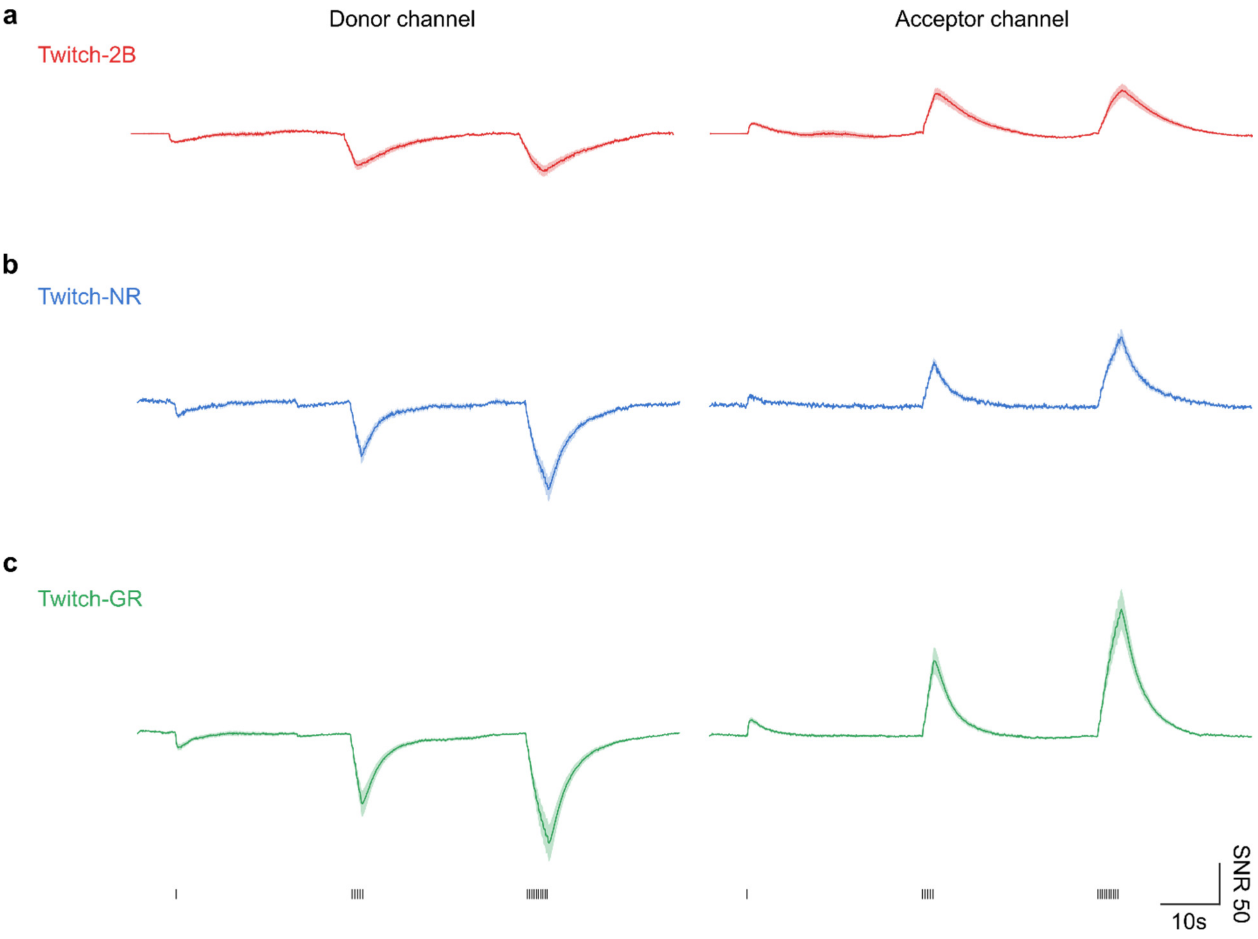

The averaged donor and acceptor SNR responses of neurons expressing **(a)** Twitch-2B, **(b)** Twitch-GR, and **(c)** Twitch-NR when responding to one, five, or ten action potentials. Light lines and transparent color regions indicate the mean response ± s.e.m. ($n \geq 10$ neurons for each sensor). Black vertical lines below each trace indicate the time of electrical stimulation.

Supplementary Figure 9 – Twitch-GR outperformed Twitch-2B in Total SNR when detecting action potentials.

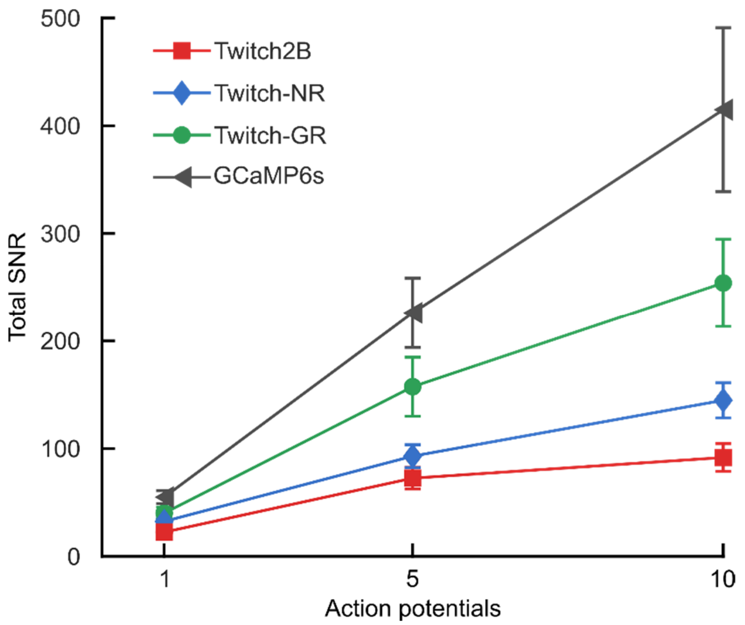

The relationship between the peak total SNR and the number of the action potentials fired by neurons expressing Twitch-2B, Twitch-GR, Twitch-NR, and GCaMP6s (error bars are s.e.m., *n* ≥ 10 neurons for each sensor).

Supplementary Figure 10 – mScarlet-TnC3-cpGFP accurately detected action potentials in cultured hippocampal neurons.

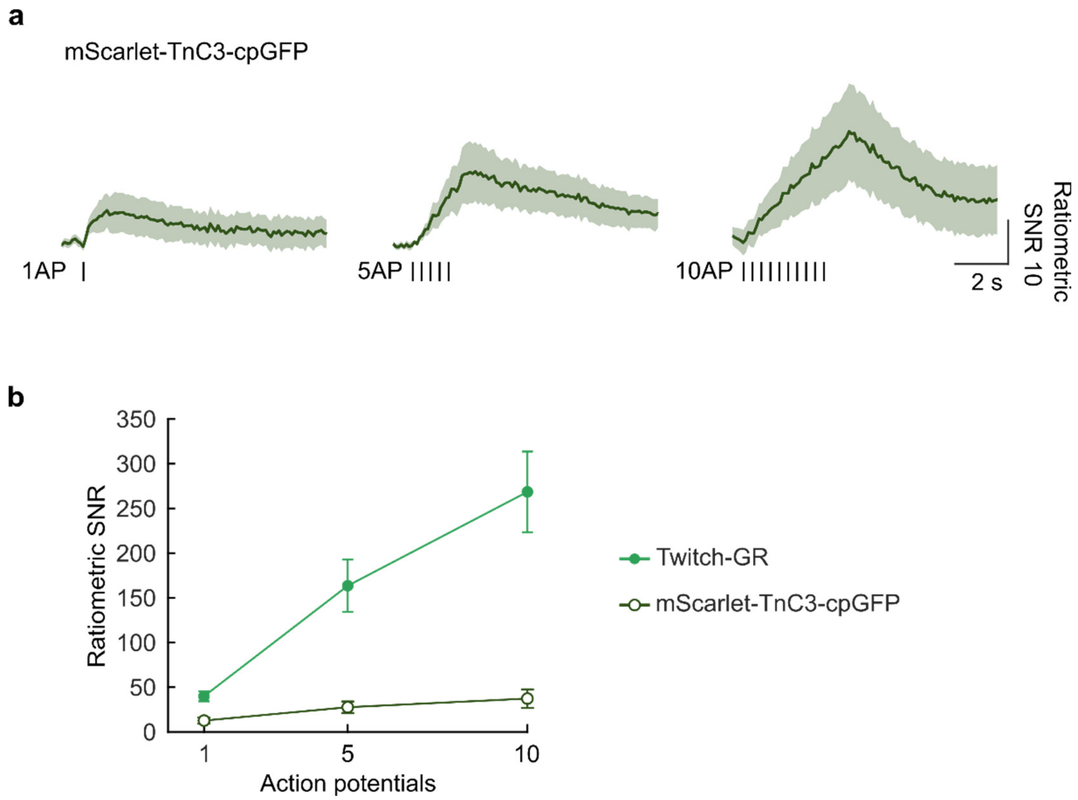

(a) The average ratiometric SNR response from neurons expressing Twitch-GR and mScarlet-TnC3-cpGFP when firing one, five, and ten action potentials, respectively. Solid lines and transparent color regions indicate the mean response ± s.e.m. (*n* = 8 neurons for each sensor). Black dashes indicate the time of the electrical stimulation. **(b)** Comparison of the ratiometric SNR in response to action potentials from neurons expressing mScarlet-TnC3-cpGFP or Twitch-GR (error bars are s.e.m., *n* = 8 neurons for mScarlet-TnC3-cpGFP and *n* = 11 neurons for Twitch-GR).

Supplementary Figure 11 – Twitch-GR and Twitch-NR sensors accurately detected action potentials from small compartments of cultured hippocampal neurons.

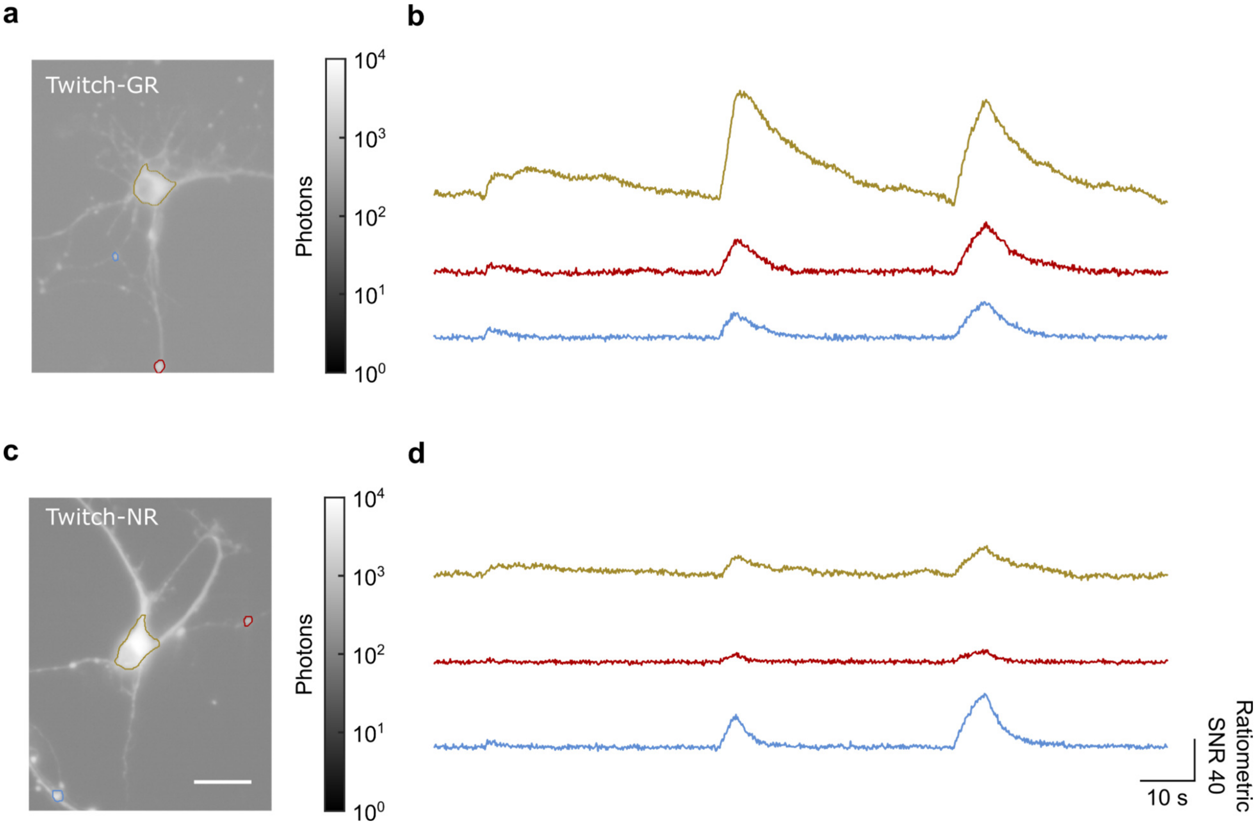

(a) Donor fluorescence of a cultured neuron expressing Twitch-GR. Red and blue regions indicate small axonal or dendritic compartments of cultured neurons, while yellow regions indicate the soma. **(b)** Ratiometric SNR responses from color coordinated compartments of the same neuron shown in panel (a) to 1, 5, and 10 electrically stimulated action potentials. **(c)** Donor fluorescence of a cultured neuron expressing Twitch-NR. Colored regions match that of panel (a). **(d)** Ratiometric SNR responses from color coordinated compartments of the same neuron shown in panel (c) to 1, 5, and 10 electrically stimulated action potentials. The scale bar is 50 μm.

Supplementary Figure 12 – Single exponential rise and fall dynamics fit the action potential response of sensors expressed in cultured neurons.

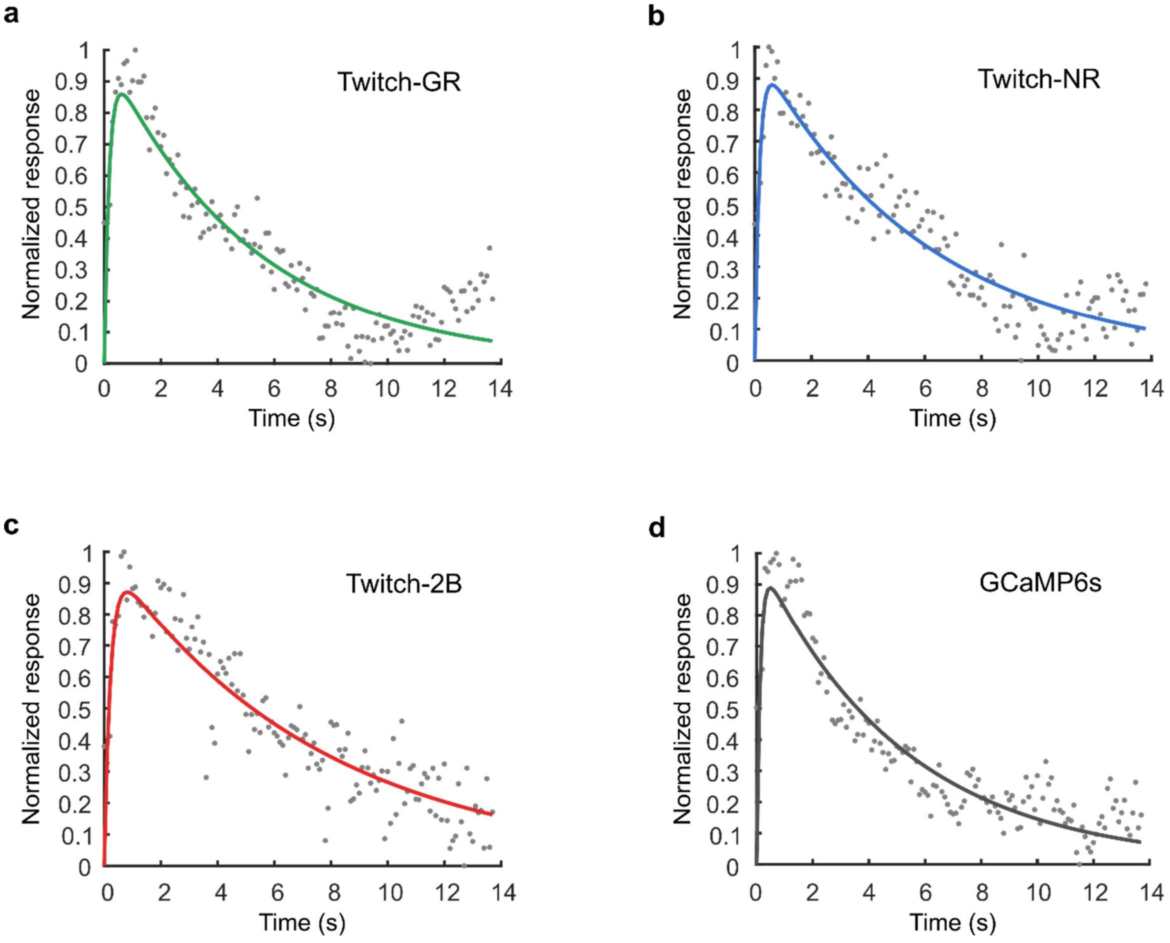

For **(a)** Twitch-GR, **(b)** Twitch-NR, **(c)** Twitch-2B, and **(d)** GCaMP6s, data points indicate the temporal response on single trials, while lines indicate the fit to dynamics that follow first-order exponential kinetics for both the rise and fall portions of the transient.

Supplementary Figure 13 – The brightness of layer 2/3 neurons expressing Twitch-GR was significantly higher than the brightness of layer 2/3 neurons expressing Twitch-2B.

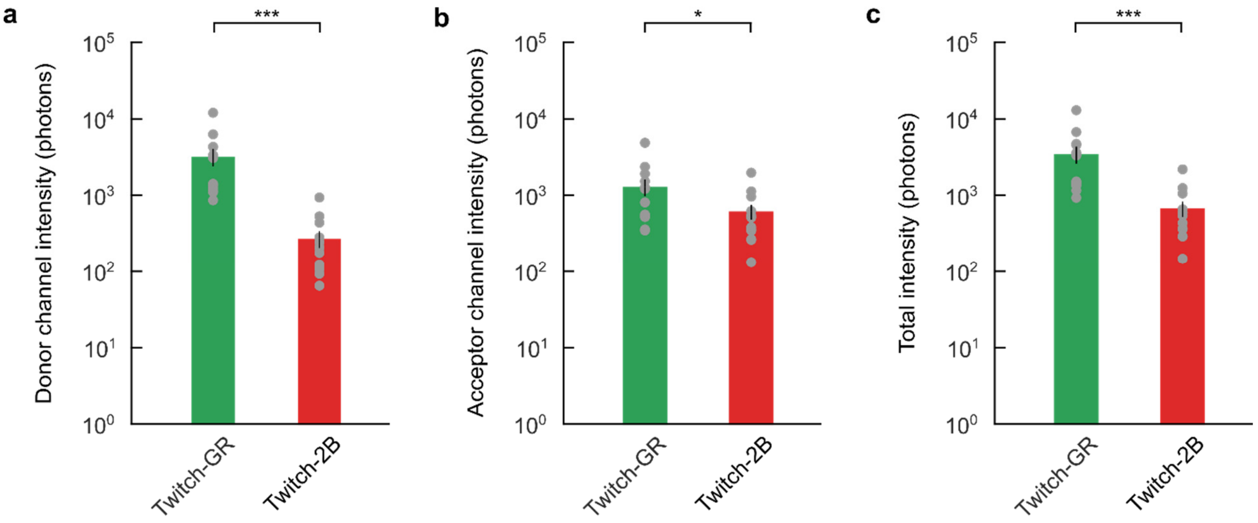

Comparisons between Twitch-GR's and Twitch-2B's **(a)** Donor channel intensity, **(b)** acceptor channel intensity, and **(c)** total intensity, imaged by two-photon microscopy. The images were obtained at matched depths from 150 μm to 300 μm and excitation powers from 20 mW to 50 mW. Intensities from individual channels were the sum of photons collected from regions of interest containing individual neurons, totaled over 4 successive frames imaged at 44 Hz. The total intensity was the sum of the individual channel intensities in quadrature. All emission intensities were normalized as if the excitation power were 50 mW. Error bars are s.e.m., $n \geq 10$ neurons for each sensor. All statistical tests were the two-sided Wilcoxon rank-sum test, $p = 1.4 \times 10^{-5}$, 0.02, and 9.4×10^{-5} for comparisons on the donor channel, acceptor channel, and total intensity, respectively. * signifies $p < 0.05$, *** signifies $p < 0.001$. Gray dots indicate individual data points.

Supplementary Figure 14 | Layer 5 was visible during *in vivo* imaging of the mouse visual cortex expressing Twitch-GR, but not visible during the imaging of the mouse visual cortex expressing Twitch-2B.

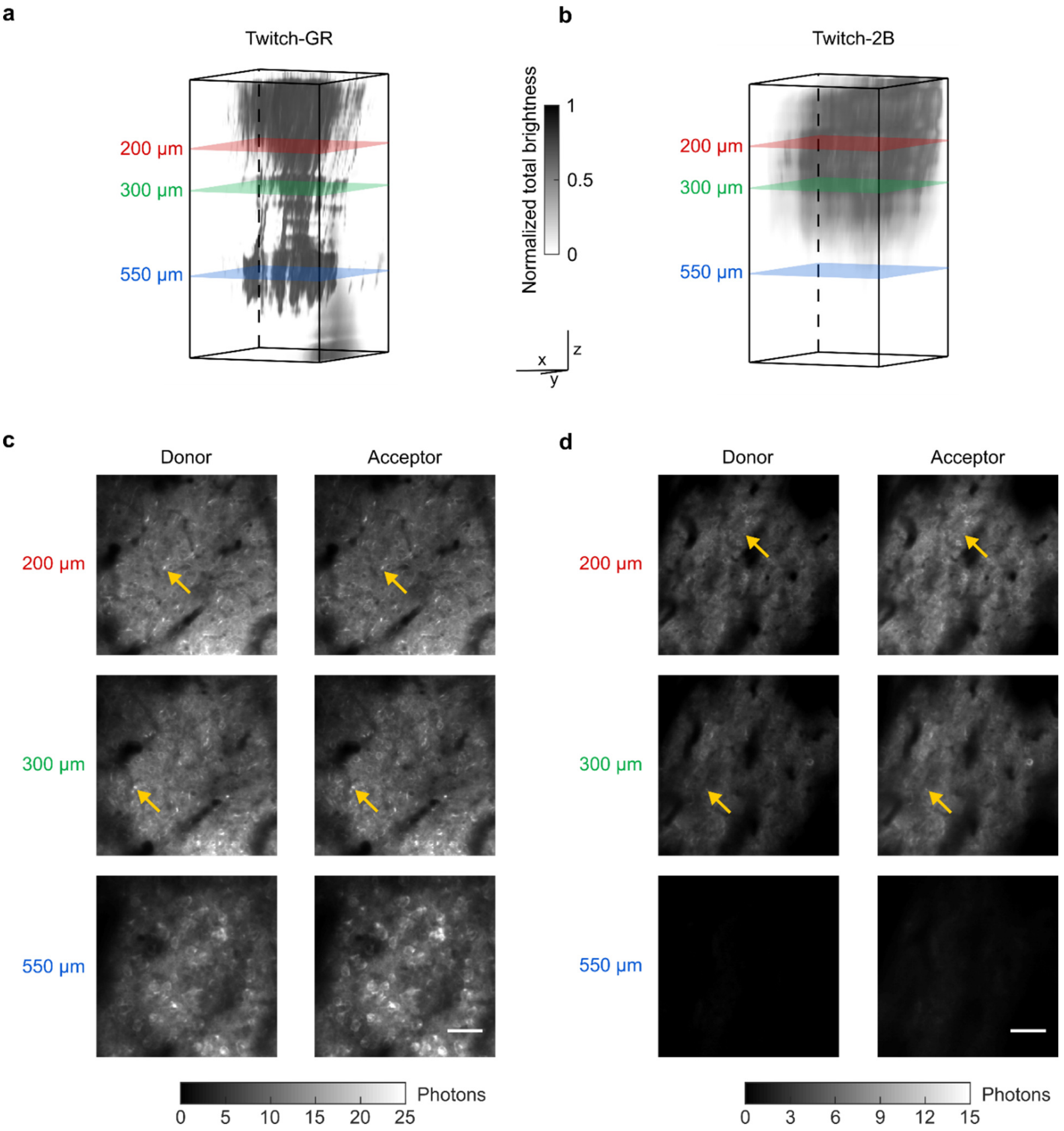

Three-dimensional stacks imaged from the surface of the brain down to a depth of 600 μm for **(a)** a representative mouse expressing Twitch-GR in the visual cortex and **(b)** a representative mouse expressing Twitch-2B in the visual cortex. The stacks show various neural structures through the cortical layers of V1. The excitation power varied from 20 mW to 100 mW to compensate for excess tissue scattering at deeper layers, but was matched at the same imaging depths between

samples. The image at each depth was normalized to the maximum brightness of that image. Scale bars are 100 μm in the *x*, *y*, and *z* directions. The donor and acceptor fluorescence intensities at the depths of 200 μm, 300 μm, and 550 μm for the same **(c)** Twitch-GR mouse and **(d)** Twitch-2B mouse helped characterize the imaging fidelity of the stacks. These individual images report neurons through multiple cortical layers and apical projections of the layer 5 neurons within layer 2/3 (*yellow arrows*). When imaging the mouse expressing Twitch-GR, images from cortical layer 2/3 (200 μm, 300 μm) and layer 5 (550 μm) showed clear neurons. When imaging the mouse expressing Twitch-2B, images from layer 5 were dim. The excitation powers that imaged the 200 μm, 300 μm, and 550 μm layers were 20 mW, 50 mW, and 100 mW, respectively. All images averaged 200 frames. The scale bars are 50 μm.

Supplementary Figure 15 | Sub-cellular structures labeled by Twitch-GR were captured higher fidelity than sub-cellular structures labeled by Twitch-2B over multiple depths.

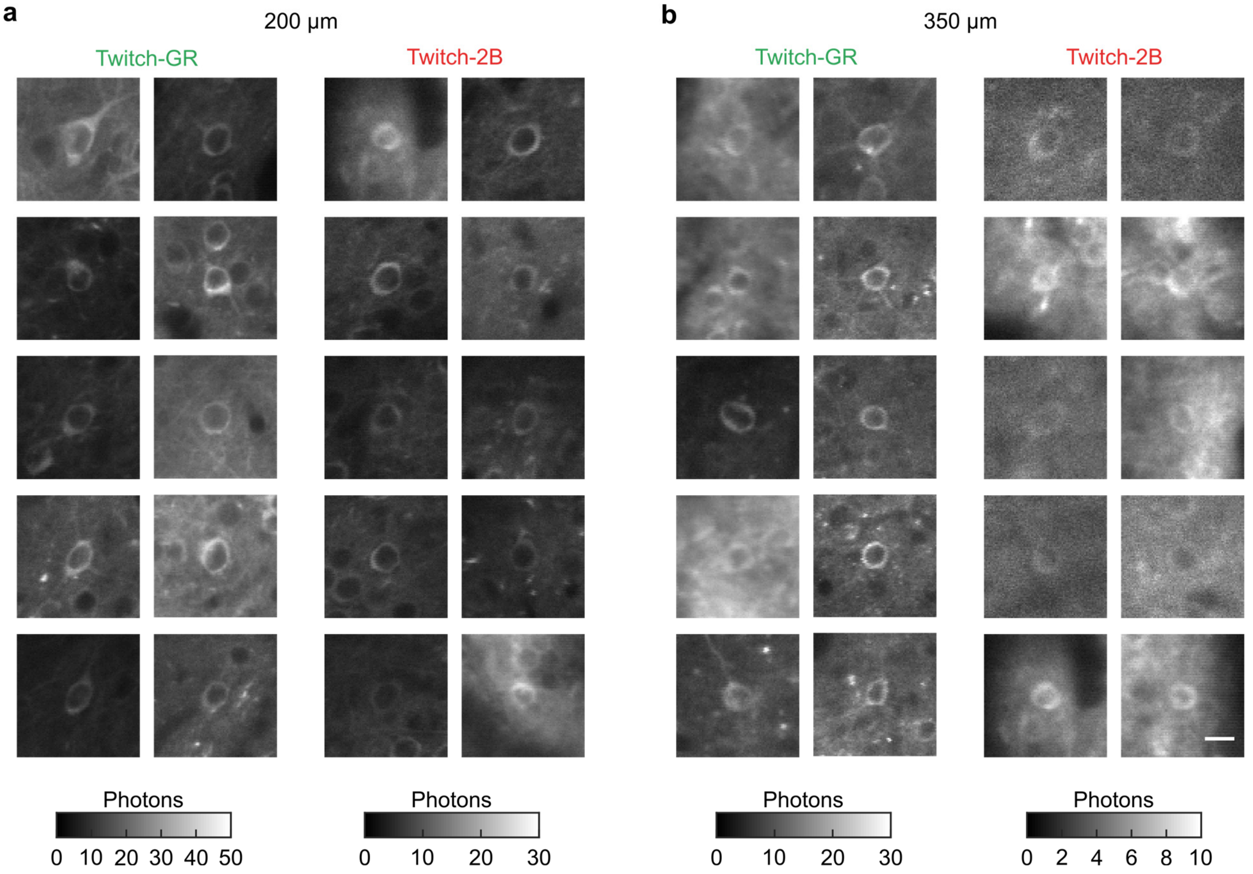

The total fluorescence images of individual neurons expressing Twitch-2B and Twitch-GR at a depth of **(a)** ~200 µm and **(b)** ~350 µm demonstrate that Twitch-GR captured spatial structures with high fidelity. When performing power-matched imaging at both depths, the brightness of neurons expressing Twitch-GR was higher than the brightness of neurons expressing Twitch-2B. At a depth of 200 µm, images of neurons expressing Twitch-2B or Twitch-GR showed a defined ring, validating the sensor localization to the neuron cytoplasm. At a depth of 350 µm, the cytoplasm of neurons expressing Twitch-2B was blurred more than the cytoplasm of neurons expressing Twitch-GR. Each image was the average of 100 imaging frames. The scale bar is 10 µm. The excitation powers that imaged the 200 µm and 350 µm layers were 20 mW and 50 mW, respectively.

Supplementary Fig. 16 | Imaging of GCaMP6s in the cortex produced transients, but were photon-starved.

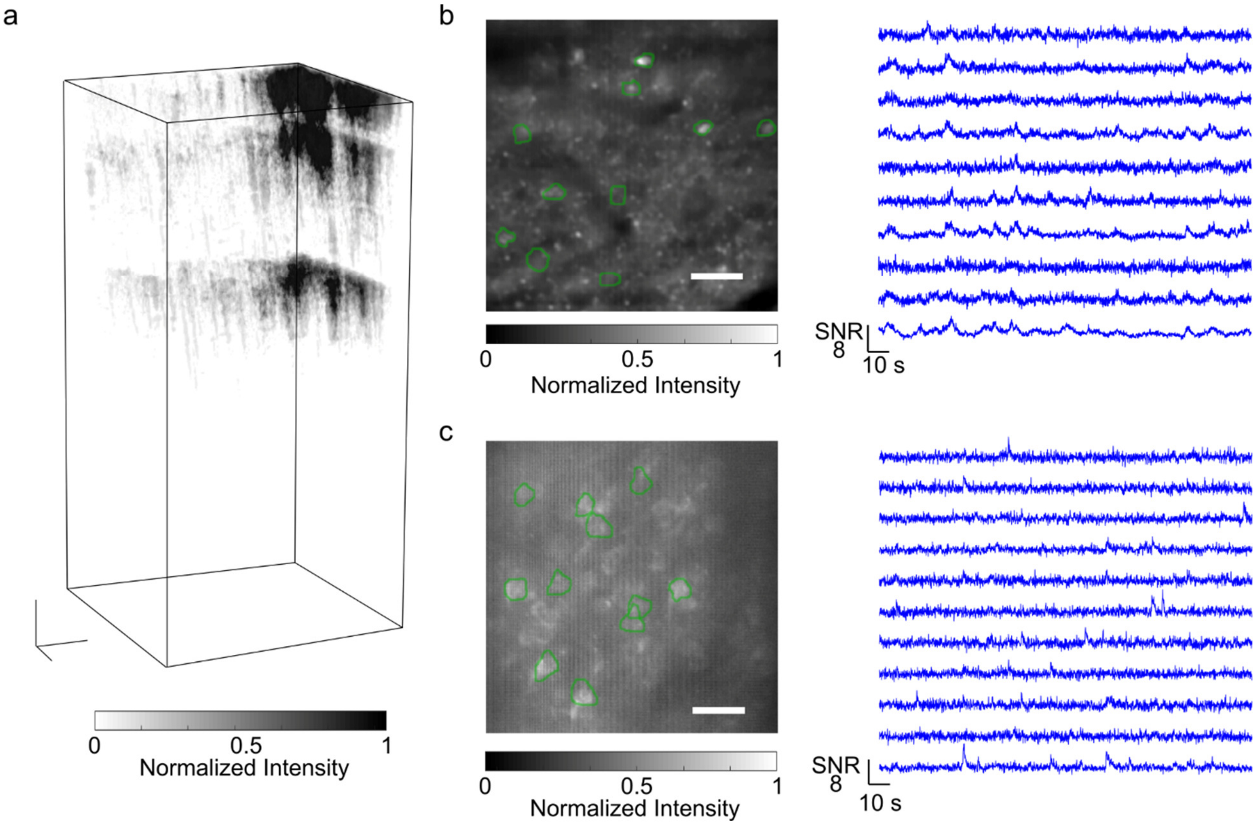

(a) Three-dimensional stacks imaged from the brain surface down to 600 µm depth from a mouse expressing GCaMP6s. The *z* stack (top) showed the entire cortical structure. The three-dimensional scale bars for the top panel are 100 µm along each of the three axes. Excitation intensities were power matched to the powers in Fig. 4e. **(b)** *Left*: Representative mean image from 200 µm deep in the cortex averaged over 400 frames. The illumination intensity was 50 mW, similar to that used for our ratiometric sensors at the same depth. Neurons highlighted for temporal analysis are highlighted by the green outlines. The scale bar is 30 µm. *Right*: Fluorescence traces from the highlighted neurons have qualitatively similar SNR as the ratiometric SNR of Fig. 4c. **(c)** *Left*: Representative mean image from 500 µm deep in the cortex averaged over 400 frames. The illumination intensity was 200 mW, higher than that used for our ratiometric sensors at the same depth. Neurons highlighted for temporal analysis are highlighted by the green outlines. The scale bar is 30 µm. *Right*: Fluorescence traces from the highlighted neurons have qualitatively similar SNR as the ratiometric SNR of Fig. 4h.

Supplementary Table 1 - Amino acid sequences of linkers between the TnC calcium sensing domain and mScarlet or between the TnC calcium sensing domain and the GFP-analogs. 'O' indicates the tested variants, and '-' indicates the untested variant. Previous study reported that amino acid sequences VADA/DA and PIPY/PLAEL respectively linking the donor and acceptor fluorescent proteins to the calcium sensing domain optimized ratiometric responses to calcium concentration change [1-2]. We employed with these linkers and truncated versions of these linkers in our sensors to optimize the FRET response of our sensors.

Linker-GFP / Linker-mScarlet	PLAEL	PIPY	PLA	PL
VADA	O	O	O	O
ADA	O	O	O	O
DA	O	O	O	O
D	O	O	O	O
A	-	O	O	O

Supplementary Table 2 | Amino acid sequence of Twitch-GR, Twitch-NR, Twitch-CiR, Twitch-ClR and Twitch-VR.

Sensors	Amino acid sequence
Twitch-GR	MLQNELALKLAGLDINKTGGGSVSKGEAVIKEFMRFKVHMEGSMNGHEFEIEGEGEGRPYEG<br>TQTAKLKVTKGGPLPFSWDILSPQFMYGSRAFTKHPADIPDYYKQSFPEGFKWERVMNFEDG<br>GAVTVTQDTSLEDGTLIYKVKLRGTNFPPDGPVMQKKTMGWEASTERLYPEDGVLKGDIKMAL<br>RLKDGGRYLADFKTTYKAKKPVQMPGAYNVDRKLDITSHNEDYTVVEQYERSEGRHSDASEE<br>ELSECFRIFDFDGNGFIDREEFGDIIRLTGEQLTDEDVDEIFGDSDTDKNGRIDFDEFLKMVENV<br>QPLASVQLADHYQQNTPIGDGPVLLPDNHYLSTQSKLSKDPNEKRDHMVLLEFVTAAGITLGM<br>DELYKGGTGGSMVSKGEELFTGVVPILVELDGDVNGHKFSVRGEGEGDATNGKLTLKFICTTG<br>KLPVPWPTLVTTLTYGVQCFSRYPDHMKQHDFFKSAMPEGYVQERTISFKDDGTYKTRAEVK<br>FEGDTLVNRIELKGIDFKEDGNILGHKLEYNFNSHNVYITADKQKNGIKANFKIRHNVEDG*
Twitch-NR	MLQNELALKLAGLDINKTGGGSVSKGEAVIKEFMRFKVHMEGSMNGHEFEIEGEGEGRPYEG<br>TQTAKLKVTKGGPLPFSWDILSPQFMYGSRAFTKHPADIPDYYKQSFPEGFKWERVMNFEDG<br>GAVTVTQDTSLEDGTLIYKVKLRGTNFPPDGPVMQKKTMGWEASTERLYPEDGVLKGDIKMAL<br>RLKDGGRYLADFKTTYKAKKPVQMPGAYNVDRKLDITSHNEDYTVVEQYERSEGRHSDASEE<br>ELSECFRIFDFDGNGFIDREEFGDIIRLTGEQLTDEDVDEIFGDSDTDKNGRIDFDEFLKMVENV<br>QPLANRYRSTARTTYTFAKPMAANYLKNQPMYVFRKTELKHSMTELNFKEWQKAFTDMMGM<br>DELYKGGTGGSGGGGSMVSKGEEDNMASLPATHELHIFGSINGVDFDMVGQGTGNPNDGYE<br>ELNLKSTKGDLQFSPWILVPHIGYGFHQYLPYPDGMSPFQAAMVDGSGYQVHRTMQFEDGAS<br>LTVNYRYTYEGSHIKGEAQVMGTGFPADGPVMTNTLTAADWCMSKKTYPNDKTIISTFKWSYT<br>TVNG*
Twitch-CiR	MLQNELALKLAGLDINKTGGGSVSKGEAVIKEFMRFKVHMEGSMNGHEFEIEGEGEGRPYEG<br>TQTAKLKVTKGGPLPFSWDILSPQFMYGSRAFTKHPADIPDYYKQSFPEGFKWERVMNFEDG<br>GAVTVTQDTSLEDGTLIYKVKLRGTNFPPDGPVMQKKTMGWEASTERLYPEDGVLKGDIKMAL<br>RLKDGGRYLADFKTTYKAKKPVQMPGAYNVDRKLDITSHNEDYTVVEQYERSEGRHSDASEE<br>ELSECFRIFDFDGNGFIDREEFGDIIRLTGEQLTDEDVDEIFGDSDTDKNGRIDFDEFLKMVENV<br>QPLAGVQLADHYQQNTPIGDGPVLLPDNHYLSYQSKLSKDPNEKRDHMVLLEFVTAAGITLGM<br>DELYKGGTGGSMVSKGEELFTGVVPILVELDGDVNGHKFSVRGEGEGDATNGKLTLKFICTTG<br>KLPVPWPTLVTTLGYGLMCFARYPDHMKQHDFFKSAMPEGYVQERTIFFKDDGNYKTRAEVK<br>FEGDTLVNRIELKGIDFKEDGNILGHKLEYNYNSHNVYIMADKQKNGIKANFKIRHNIEDG*
Twitch-ClR	MLQNELALKLAGLDINKTGGGSVSKGEAVIKEFMRFKVHMEGSMNGHEFEIEGEGEGRPYEG<br>TQTAKLKVTKGGPLPFSWDILSPQFMYGSRAFTKHPADIPDYYKQSFPEGFKWERVMNFEDG<br>GAVTVTQDTSLEDGTLIYKVKLRGTNFPPDGPVMQKKTMGWEASTERLYPEDGVLKGDIKMAL<br>RLKDGGRYLADFKTTYKAKKPVQMPGAYNVDRKLDITSHNEDYTVVEQYERSEGRHSDASEE<br>ELSECFRIFDFDGNGFIDREEFGDIIRLTGEQLTDEDVDEIFGDSDTDKNGRIDFDEFLKMVENV<br>QPLASVQLADHYQQNTPIGDGPVLLPDNHYLSHQSKLSKDPNEKRDHMVLLEFVTAAGITHGM<br>DELYKGGTGGSMVSKGEELFTGVVPILVELDGDVNGHKFSVRGEGEGDATNGKLTLKFICTTG<br>KLPVPWPTLVTTFGYGVACFSRYPDHMKQHDFFKSAMPEGYVQERTISFKDDGTYKTRAEVK<br>FEGDTLVNRIELKGIDFKEDGNILGHKLEYNFNSHYVYITADKQKNCIKANFKIRHNVEDG*
Twitch-VR	MLQNELALKLAGLDINKTGGGSVSKGEAVIKEFMRFKVHMEGSMNGHEFEIEGEGEGRPYEG<br>TQTAKLKVTKGGPLPFSWDILSPQFMYGSRAFTKHPADIPDYYKQSFPEGFKWERVMNFEDG<br>GAVTVTQDTSLEDGTLIYKVKLRGTNFPPDGPVMQKKTMGWEASTERLYPEDGVLKGDIKMAL<br>RLKDGGRYLADFKTTYKAKKPVQMPGAYNVDRKLDITSHNEDYTVVEQYERSEGRHSDASEE<br>ELSECFRIFDFDGNGFIDREEFGDIIRLTGEQLTDEDVDEIFGDSDTDKNGRIDFDEFLKMVENV<br>QPLAGVQLADHYQQNTPIGDGPVLLPDNHYLSYQSKLSKDPNEKRDHMVLLEFVTAAGITLGM<br>DELYKGGTGGSMVSKGEELFTGVVPILVELDGDVNGHKFSVRGEGEGDATNGKLTLKLICTTG<br>KLPVPWPTLVTTLGYGLMCFARYPDHMKQHDFFKSAMPEGYVQERTIFFKDDGNYKTRAEVK<br>FEGDTLVNRIELKGIDFKEDGNILGHKLEYNYNSHNVYITADKQKNGIKANFKIRHNIEDG*

Supplementary Table 3 | Properties of sensor variants.

	Donor		Acceptor		K_d (nM)
	Brightness[a]	$T_{Photobleaching}$ (s)	Brightness[a]	$T_{Photobleaching}$ (s)	
Twitch-GR	2.08 ± 0.30	2633 ± 246	0.79 ± 0.11	3120 ± 315	150
Twitch-NR	2.53 ± 0.43	2172 ± 287	0.81 ± 0.13	2388 ± 500	610
Twitch-CiR	3.26 ± 0.46	494 ± 30	1.00 ± 0.16	889 ± 89	1150
Twitch-VR	1.74 ± 0.21	739 ± 50	0.61 ± 0.08	1444 ± 93	1820
Twitch-ClR	1.65 ± 0.28	1323 ± 137	0.59 ± 0.10	2604 ± 311	1240
Twitch-2B	1.00 ± 0.09	1544 ± 56	1.00 ± 0.10	1225 ± 62	200
Twitch-3	1.07 ± 0.10	2080 ± 184	1.53 ± 0.16	854 ± 19	-

[a] Donor and acceptor cellular brightness of cells expressing the sensor, normalized to the cellular brightness of cells expressing Twitch-2B

Supplementary Table 4 | Kinetics of sensors during *in vivo* imaging.

	Rise time τ_{on} (s)	Decay time τ_{off} (s)
Twitch-2B	1.30 ± 0.08	4.06 ± 0.30
Twitch-GR	0.95 ± 0.09	2.57 ± 0.13

Supplementary References:

[1] Thestrup, T., J. Litzlbauer, I. Bartholomaus, et al., Optimized ratiometric calcium sensors for functional in vivo imaging of neurons and T lymphocytes. Nat. Methods, 2014. 11(2): p. 175-82.

[2] Yang, J., Cumberbatch, D., Centanni, S., et al., Coupling optogenetic stimulation with NanoLuc-based luminescence (BRET) Ca++ sensing. Nat. Comm., 2016. 7: p. 13268.
