# Supplementary material for: Rational engineering of ratiometric calcium sensors with bright green and red fluorescent proteins
Source: Commun Biol. 2021 Jul 29;4:924. doi: 10.1038/s42003-021-02452-z (PMC8322158; doi:10.1038/s42003-021-02452-z)
Supplement: Supplementary file 2 — Reporting Summary [file 42003_2021_2452_MOESM2_ESM.pdf]

Corresponding author(s): Yiyang Gong
Last updated by author(s): Jun 24, 2021

Reporting Summary

Nature Research wishes to improve the reproducibility of the work that we publish. This form provides structure for consistency and transparency in reporting. For further information on Nature Research policies, see our Editorial Policies and the Editorial Policy Checklist.

Statistics

For all statistical analyses, confirm that the following items are present in the figure legend, table legend, main text, or Methods section.

n/a	Confirmed	
☐	☒	The exact sample size (*n*) for each experimental group/condition, given as a discrete number and unit of measurement
☐	☒	A statement on whether measurements were taken from distinct samples or whether the same sample was measured repeatedly
☐	☒	The statistical test(s) used AND whether they are one- or two-sided<br>*Only common tests should be described solely by name; describe more complex techniques in the Methods section.*
☒	☐	A description of all covariates tested
☒	☐	A description of any assumptions or corrections, such as tests of normality and adjustment for multiple comparisons
☐	☒	A full description of the statistical parameters including central tendency (e.g. means) or other basic estimates (e.g. regression coefficient) AND variation (e.g. standard deviation) or associated estimates of uncertainty (e.g. confidence intervals)
☐	☒	For null hypothesis testing, the test statistic (e.g. *F*, *t*, *r*) with confidence intervals, effect sizes, degrees of freedom and *P* value noted<br>*Give P values as exact values whenever suitable.*
☒	☐	For Bayesian analysis, information on the choice of priors and Markov chain Monte Carlo settings
☒	☐	For hierarchical and complex designs, identification of the appropriate level for tests and full reporting of outcomes
☒	☐	Estimates of effect sizes (e.g. Cohen's *d*, Pearson's *r*), indicating how they were calculated

Our web collection on statistics for biologists contains articles on many of the points above.

Software and code

Policy information about availability of computer code

Data collection	We used a Nikon E600FN microscope and a custom-built resonant two-photon microscope to collect data from cell culture in vitro and mouse brain in vivo.
Data analysis	We used MATLAB 2017b and ImageJ 1.52a to analyze the data.

For manuscripts utilizing custom algorithms or software that are central to the research but not yet described in published literature, software must be made available to editors and reviewers. We strongly encourage code deposition in a community repository (e.g. GitHub). See the Nature Research guidelines for submitting code & software for further information.

Data

Policy information about availability of data

All manuscripts must include a data availability statement. This statement should provide the following information, where applicable:

- Accession codes, unique identifiers, or web links for publicly available datasets
- A list of figures that have associated raw data
- A description of any restrictions on data availability

Data needed to reproduce our figures will be publicly available online upon acceptance of this manuscript.

Field-specific reporting

Please select the one below that is the best fit for your research. If you are not sure, read the appropriate sections before making your selection.

☒ Life sciences ☐ Behavioural & social sciences ☐ Ecological, evolutionary & environmental sciences

For a reference copy of the document with all sections, see nature.com/documents/nr-reporting-summary-flat.pdf

Life sciences study design

All studies must disclose on these points even when the disclosure is negative.

Sample size	For cell culture test in vitro, sample sizes were always ≥ 10 cells from 3 culture wells for each test. We totally imaged more than 1,000 cells for sensor identification in vitro. For mouse imaging in vivo, sample sizes were always 3 to 5 mice for each test. The number of mice was dependent on the yield of usable mice and the number of animals initially implanted and injected. All together, we implanted 50 mice and yield 16 usable mice.
Data exclusions	Data were not excluded from analysis.
Replication	Reported results were consistently replicated across multiple experiments with all replicates generating similar results.
Randomization	For cell culture test in vitro, we selected cells randomly for imaging. For mouse imaging in vivo, we assigned animals randomly to experimental and control groups.
Blinding	Blinding was not possible as experimental conditions were evident from the image data. Quantifications were performed using computational pipeline applied equally to all conditions and replicates for each test.

Reporting for specific materials, systems and methods

We require information from authors about some types of materials, experimental systems and methods used in many studies. Here, indicate whether each material, system or method listed is relevant to your study. If you are not sure if a list item applies to your research, read the appropriate section before selecting a response.

Materials & experimental systems

n/a	Involved in the study	
☒	☐	Antibodies
☐	☒	Eukaryotic cell lines
☒	☐	Palaeontology and archaeology
☐	☒	Animals and other organisms
☒	☐	Human research participants
☒	☐	Clinical data
☒	☐	Dual use research of concern

Methods

n/a	Involved in the study	
☒	☐	ChIP-seq
☒	☐	Flow cytometry
☒	☐	MRI-based neuroimaging

Eukaryotic cell lines

Policy information about cell lines

Cell line source(s)	HEK 293 - Takara Biosciences
Authentication	none of the cell lines were authenticated
Mycoplasma contamination	Cell lines were not tested for Mycoplasma contamination
Commonly misidentified lines (See ICLAC register)	*Name any commonly misidentified cell lines used in the study and provide a rationale for their use.*

Animals and other organisms

Policy information about studies involving animals; ARRIVE guidelines recommended for reporting animal research

Laboratory animals	For neuron culture, we used day 0 Sprague-Dawley pups (CD001 rat, Charles River Labs). For mouse imaging in vivo, we used 8 week old male mice (C57BL/J6 mouse, Jackson Laboratory).
Wild animals	We did not use wild animals.

Field-collected samples	Study did not involve field-collected samples.
Ethics oversight	We performed the rat and mouse work under the study protocol A014-18-01, as approved by the Duke Institutional Animal Care and Use Committee (IACUC).

Note that full information on the approval of the study protocol must also be provided in the manuscript.
